# Siderophore sharing protects clonal *Pseudomonas aeruginosa* biofilms from colistin

**DOI:** 10.1128/aem.00588-26

**Published:** 2026-06-17

**Authors:** Shuaitao Wang, Wei Ding, Hongzhe Peng, Lihui Ren, Bo Dong, Jinshui Lin, Weipeng Zhang

**Affiliations:** 1 MOE Key Laboratory of Evolution & Marine Biodiversity and Institute of Evolution & Marine Biodiversity, Ocean University of China 12591, Qingdao, China; 2 MOE Key Laboratory of Marine Genetics & Breeding and College of Marine Life Sciences, Ocean University of China 12591, Qingdao, China; 3 Fang Zongxi Center for Marine EvoDevo, Ocean University of China 12591, Qingdao, China; 4 School of Rehabilitation Sciences and Engineering, University of Health and Rehabilitation Sciences 652610 , Qingdao, China; 5 Shaanxi Key Laboratory of Chinese Jujube, College of Life Sciences, Yan’an University 105849, Yan'an, China; Indiana University Bloomington, Bloomington, Indiana, USA

**Keywords:** scRNA-seq, siderophore, colistin, biofilm, *Pseudomonas aeruginosa*

## Abstract

Siderophores mediate cooperative iron acquisition and stress tolerance in polymicrobial communities. However, whether and how such social roles emerge within clonal populations of a single bacterial strain remain unclear. Here, we investigate whether and how such social roles emerge within clonal populations of a single bacterial strain. Using single-cell transcriptomics, we analyzed the response of clonal *Pseudomonas aeruginosa* PAO1 biofilms to colistin and identified 13 transcriptionally distinct subpopulations, with siderophore biosynthesis genes enriched in one subpopulation. Upon colistin exposure, these genes were specifically upregulated in this subpopulation, whereas their cognate receptor genes were ubiquitously expressed across all subpopulations. Through genetic and physiological experiments, we demonstrate that siderophore sharing between the subpopulations confers cross-protection against colistin by enhancing resistance in a growth-independent manner, as well as by promoting biofilm formation and stability. Therefore, the spontaneous emergence of siderophore producers and non-producer subpopulations, along with their intercellular interaction mediated by siderophore sharing, constitutes a strategy for colistin protection.

**IMPORTANCE:**

This work provides a single-cell transcriptomic atlas of *P. aeruginosa* biofilms and reveals transcriptional heterogeneity within the clonal population. Colistin treatment markedly reshaped the subpopulation structure, with a siderophore biosynthesis-related subpopulation being significantly enriched, while siderophore receptor genes were broadly expressed across all subpopulations. This pattern of “production specialization” and “reception universalization” extends the applicability of public goods theory to clonal bacterial populations at the single-cell level and further reveals the important role of siderophore sharing in mediating cross-protection among genetically identical subpopulations. These findings deepen our understanding of the complexity of bacterial behaviors and provide potential therapeutic targets for combating biofilm-associated infections.

## INTRODUCTION

Iron is an essential yet scarcely bioavailable micronutrient in most environments, posing a fundamental challenge for bacterial survival and growth (1). To overcome this limitation, many bacteria produce and secrete siderophores, which are high-affinity iron-chelating molecules that scavenge extracellular iron and deliver it to the cell (2). For decades, the primary focus of siderophore research was on their biochemistry and their role as individual virulence factors in pathogens. However, a significant paradigm shift has occurred in recent years, reconceptualizing siderophores not merely as private goods but as classic public goods within microbial communities (3). For example, pyoverdine (PVD) and pyochelin (PCH) are iron-scavenging molecules produced by *Pseudomonas aeruginos*a, and these molecules play crucial roles in mediating interactions between *P. aeruginosa* and other microbial species in polymicrobial communities (4, 5).

Seminal work has demonstrated that the public or private nature of a siderophore is defined by the specificity of its cognate receptor (6, 7). This creates a complex social landscape of cooperation, cheating, and competition (6, 7). Genomic and experimental studies, particularly on *Pseudomonas* species, have revealed diverse social strategies: from “cooperators” that produce shareable siderophores, to “cheaters” that exploit public goods without contributing, and “loners” that use private systems (6, 7). The evolution and stability of cooperation are influenced by factors like spatial structure (e.g., biofilms) and metabolic constraints (8). Despite these significant advances, critical knowledge gaps persist. A key unanswered question is whether siderophores mediate social interactions within a clonal bacterial population, such as among genetically identical cells in a biofilm formed by a single strain. That is, can isogenic cells differ in their investment in public goods such as siderophores, thereby generating producers and non-producers even in the absence of genetic variation? If so, what is the functional significance of this differentiation in combating environmental stresses, such as fostering antibiotic resistance (i.e., the ability to grow in the presence of antibiotics) and tolerance (i.e., survival under the treatment of antibiotics)?

Single-cell transcriptomic sequencing (scRNA-seq) has recently emerged as a transformative tool for dissecting functional heterogeneity within bacterial populations (9–12). By enabling the identification of specialized cellular subtypes, this approach has revolutionized our understanding of bacterial physiology, particularly in revealing subpopulations critical for host infection (12). Concurrently, biofilms are recognized for their multicellular nature, often exhibiting cooperative behaviors such as siderophore-mediated interactions across species or strains (13). In addition, *P. aeruginosa* PAO1 serves as a paradigm for studying biofilm biology due to its extraordinary adaptive plasticity, and clinically, it ranks among the “ESKAPE” pathogens (14).

Building on the above-mentioned knowledge gaps and foundations, here, we applied scRNA-seq to investigate the transcriptomic response of *P. aeruginosa* PAO1 biofilms to colistin, a last-resort antibiotic against multidrug-resistant gram-negative pathogens. Exposure to colistin triggered a marked, subpopulation-specific upregulation of key siderophore biosynthesis genes, effectively partitioning the clonal biofilm into distinct “producer” and “non-producer” subpopulations. This finding prompted us to further probe the mechanisms underlying siderophore-mediated interactions between these subpopulations that protect the whole population against colistin.

## RESULTS

### Single-cell transcriptional heterogeneity in PAO1 biofilms

**Fig 1:**
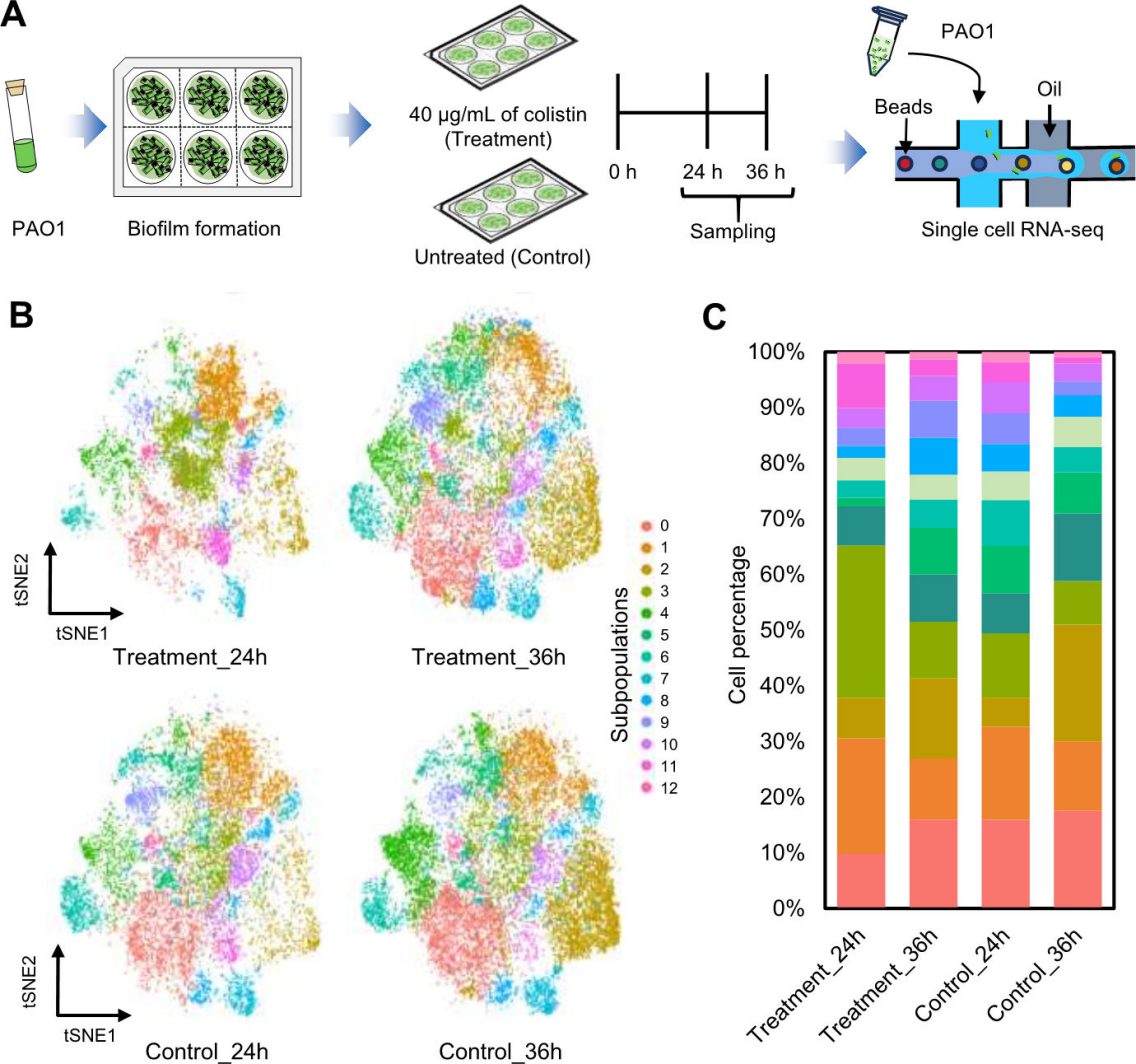
scRNA-seq reveals single-cell transcriptional landscape of PAO1 biofilms. (**A**) Experimental workflow of colistin treatment and sampling for scRNA-seq. (**B**) t-SNE projection of gene expression matrices that divide the cells into 13 subpopulations. Matrices from treatment/control groups at each time point were integrated using CCA to remove batch effects. Cells were clustered after dimensionality reduction. (**C**) Cell number percentage distribution across subpopulations in the scRNA-seq data sets.

To obtain samples for scRNA-seq, the 24-h biofilms of PAO1 formed on the bottom surface of a six-well plate were treated with colistin for different periods (i.e., 24 and 36 h), while untreated biofilm was used as the control (Fig. 1A). Then, scRNA-seq based on random primers and droplet microfluidics technology was employed to explore the transcriptional response of PAO1 biofilms to colistin treatment (Fig. 1A). After sequencing and preliminary data analysis, a similar number of cells were obtained for treatment and control groups at each time point. In the 24-h group, control and treated biofilms had 8,322 and 9,157 cells, respectively (Fig. S1). In the 36-h group, control and treatment samples had 13,879 and 13,364 cells, respectively (Fig. S1). These results suggest that for each time point, control and treated samples were comparable. More details of scRNA-seq data sets are given in Table S1.

A gene expression matrix was constructed by combining the scRNA-seq data of all four samples. Subsequently, unique molecular identifiers (UMIs) and feature RNA molecules were identified. For all samples, mRNA UMIs accounted for over 80% of all the UMIs (Fig. S2). Using a gene expression matrix constructed from the pooled four samples, we performed clustering analysis and t-distributed stochastic neighbor embedding (t-SNE) dimensionality reduction. The resulting cell clusters were then visualized separately for each of the four biofilm samples. As a result, transcriptional heterogeneity was detected for all samples, with the presence of 13 distinct cell clusters (i.e., subpopulations) (Fig. 1B). For each time point, treatment with colistin changed the relative abundance of certain subpopulations (Fig. 1C), implying that distinct subpopulations may have different responses to colistin.

### Siderophore biosynthesis is confined to specific subpopulations

By performing “FindAllMarkers” analysis across all clusters, we further found that each cell subpopulation exhibited distinct functional characteristics (Fig. S3). For example, clusters 4 and 7 were enriched in flagellar and chemotaxis genes (e.g., PA1092 encoding B-type flagellin FliC), corresponding to motile subpopulations (Fig. S3). Cluster 6 contained numerous genes encoding heat shock proteins and molecular chaperones (e.g., PA4761 encoding the chaperone protein DnaK), indicating a stress-response subpopulation (Fig. S3). Cluster 11 displayed a unique transcriptional profile dominated by siderophore biosynthesis genes (e.g., PA2386 encoding PvdA), including those for the biosynthesis of PVD and PCH (Fig. S3). These results reveal the subpopulation differentiation in PAO1 biofilms, where specific subpopulations increased in proportion as more cells adopted distinct expression patterns in response to colistin.

**Fig 2:**
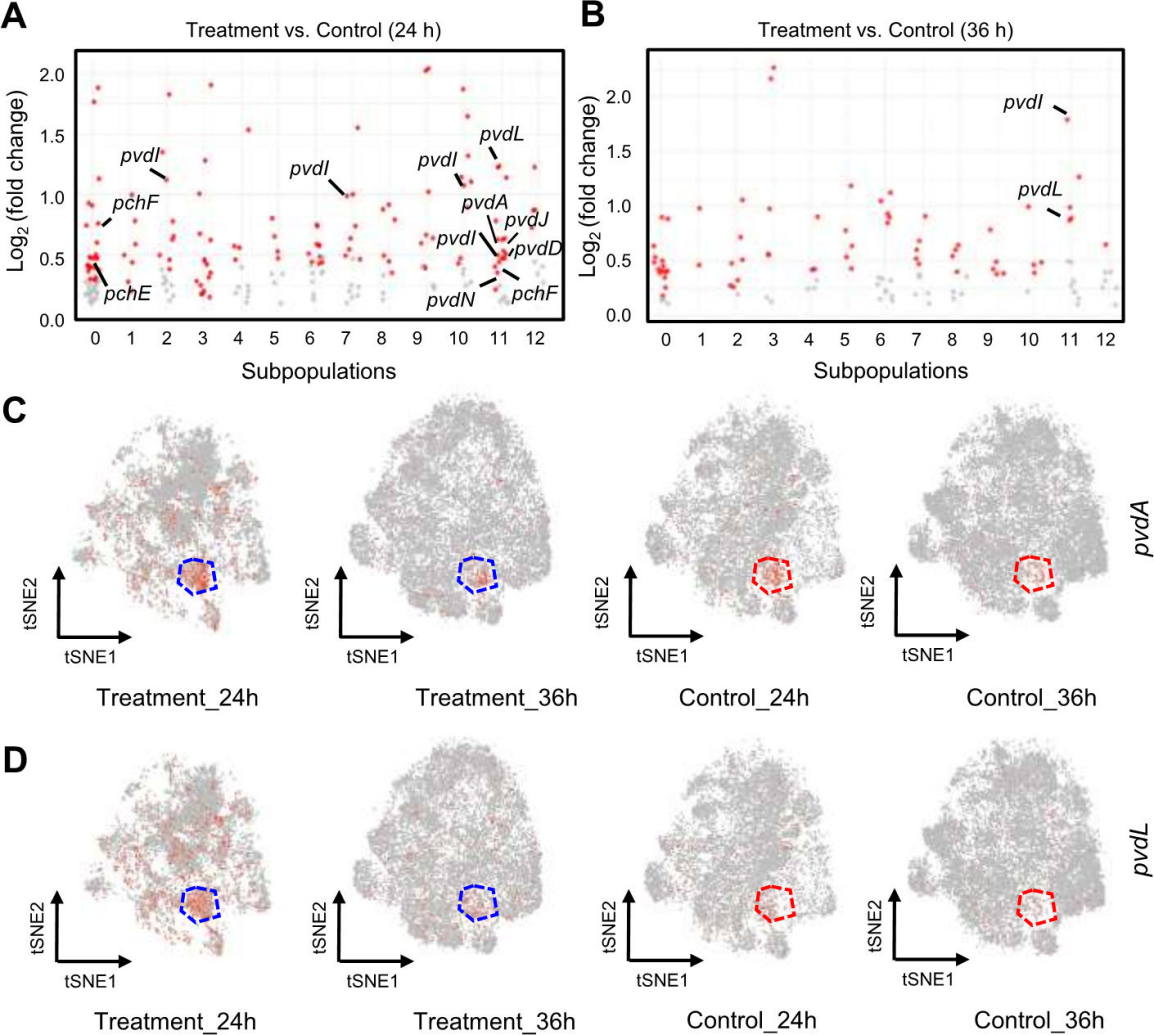
Single-cell visualization of siderophore biosynthesis gene expression. (**A**) Manhattan plots displaying differentially expressed genes between Treatment_24h vs. Control_24h. (**B**) Manhattan plots displaying differentially expressed genes between Treatment_36h vs. Control_36h. Significance thresholds were set as Log₂ FoldChange > 0.1, *P* value < 0.05, and detection in at least 25% of cells in either the treatment or control group within each subpopulation. Each point represents a gene; red dots indicate significantly upregulated genes, whereas gray dots indicate genes without significant upregulation. (**C**) t-SNE projections visualizing expression patterns of *pvdA*. (**D**) t-SNE projections visualizing expression patterns of *pvdL*. Subpopulation 11, which exhibits differential cell counts expressing *pvdA* and *pvdL* between treatment and control conditions, is highlighted with a dashed circle.

Statistical analysis (Wilcoxon rank-sum test) was further conducted to reveal the differentially expressed genes between treatment and control groups, for each subpopulation at each time point. The full list of the differentially expressed genes as revealed by the scRNA-seq is given in Table S2. In particular, the genes involved in siderophore biosynthesis, including those for PVD (e.g., *pvdA*, *pvdD*, *pvdI*, and *pvdL*) and PCH (e.g., *pchE* and *pchF*), showed significant differential expression. At 24 and 36 h after colistin treatment, the PVD biosynthesis genes *pvdA* and *pvdL* were specifically and highly expressed in subpopulation 11, as reflected by both an increased number of expressing cells and higher expression levels per cell (Fig. 2A and B; Fig. S4A and B). In contrast, although the PCH biosynthesis genes *pchE* and *pchF* were upregulated in subpopulations 0 and 11 at 24 h post-treatment, they exhibited a relatively lower proportion of highly expressing cells and overall weaker expression intensity compared to the PVD-related genes, particularly at 36 h (Fig. 2A and B; Fig. S4C and D). These findings are consistent with previous reports showing a shift from PCH to PVD production as cell density increases (15).

To provide a more intuitive visualization, we highlighted the expression of *pvdA* and *pvdL* in the t-SNE projections of subpopulation 11 (Fig. 2C and D). *pvdA* encodes L-ornithine N5-oxygenase, which is required for peptidic side-chain synthesis, whereas *pvdL* encodes a non-ribosomal peptide synthetase involved in both pyoverdine precursor assembly and chromophore precursor formation. Consistent with this, the relative abundance of this cell subpopulation showed a remarkable increase at both time points after colistin treatment (Fig. 1C). This subpopulation-specific transcriptional response may represent an important basis for the development of colistin resistance. We then wondered whether the expression of genes encoding receptors of PVD and PCH was also confined to certain subpopulations of the PAO1 biofilms. The expression profiles of two key outer membrane receptors *fpvA* (for PVD) and *fptA* (for PCH) were examined based on the scRNA-seq data. Both genes were expressed in all subpopulations, without significant difference between the treatment and the control samples (Fig. S5). At 24 h, *fptA* expression in cluster 2 of the control sample was even higher than that in the treatment sample (Fig. S5). These results suggest that in contrast to the siderophore biosynthesis genes, genes encoding siderophore receptors tend to be evenly expressed among all the subpopulations.

### Fluorescence *in situ* hybridization (FISH) confirms the scRNA-seq results

**Fig 3:**
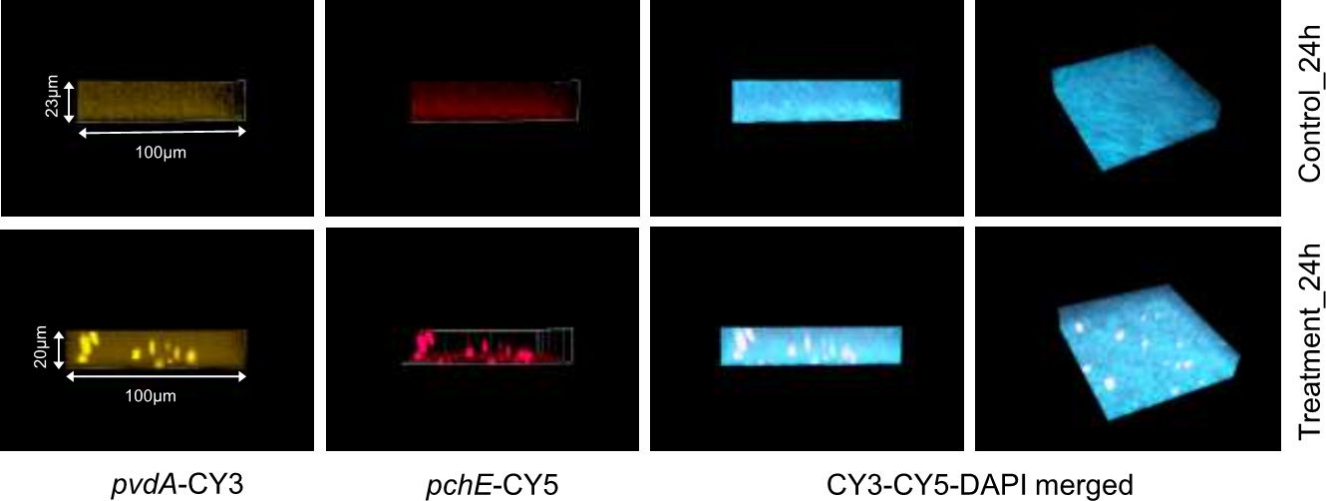
FISH analysis showing co-localization of *pvdA* and *pchE* in biofilms. A CY3-labeled probe (yellow) targets *pvdA*, a CY5-labeled probe (red) detects *pchE*, and DAPI (blue) stains all bacterial cells. The biofilms were grown statically for 24 h before treatment and then incubated for another 24 h.

To confirm the results of the scRNA-seq, fluorescence *in situ* hybridization (FISH) was performed, targeting *pvdA* and *pchE* genes in the 24-h biofilm samples. The *pvdA* and *pchE* genes were hybridized using a *pvdA*-Cy3 probe and a *pchE*-Cy5 probe, respectively, followed by CLSM. The Cy3 and Cy5 signals detected in the treatment sample were stronger than those in the control (Fig. 3). Cells with Cy3 and Cy5 signals formed several masses, which were distributed across the bottom and top surfaces of treatment samples (Fig. 3). Moreover, the majority of the cells with Cy3 and Cy5 signals overlapped with each other (Fig. 3), suggesting that they are expressed in the same subpopulations. These FISH-based observations confirm the results of the scRNA-seq, that is, the expression of siderophore biosynthesis genes is confined to certain but not all subpopulations.

### Siderophores promote colistin resistance in a growth-independent way

**Fig 4:**
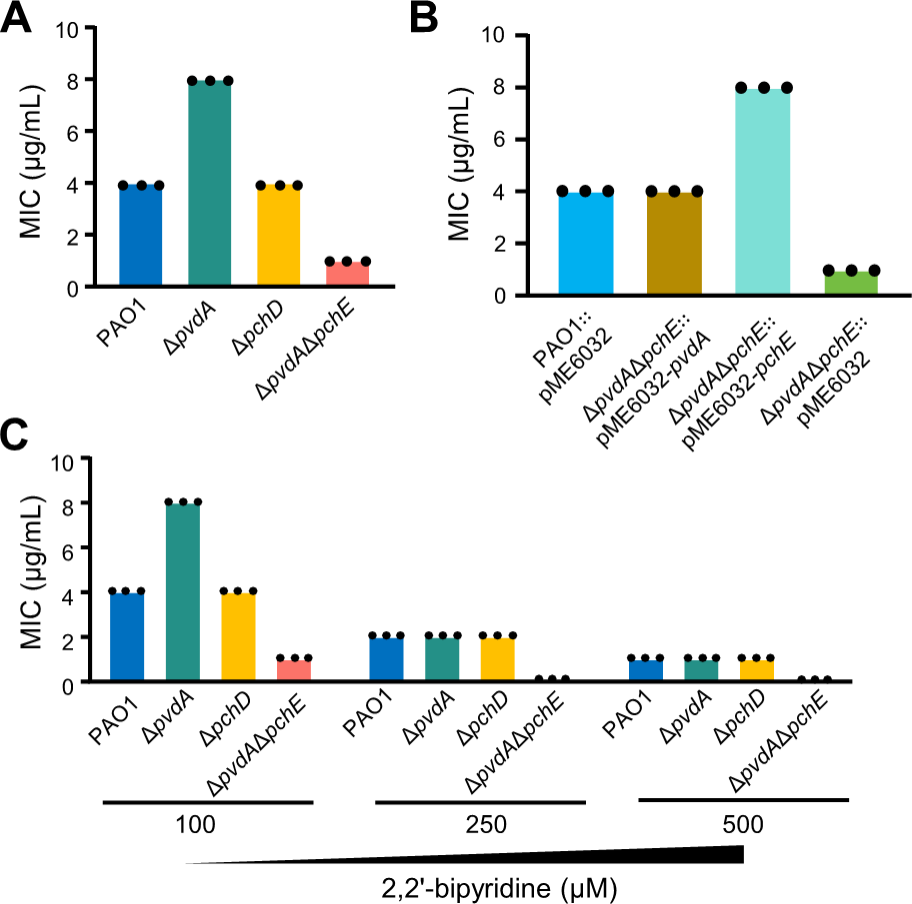
Siderophores are involved in colistin resistance. (**A**) Colistin MICs for wild-type PAO1 and siderophore biosynthesis-deficient mutants in iron-limited minimal medium. (**B**) Colistin MICs for genetically complemented strains. (**C**) Effect of exogenous iron chelator 2,2'-bipyridine on colistin MICs. In all figures, data represent three biological replicates, with the black dots indicating individual data points. Because the three values derived from the three biological replicates are identical, no error bars are shown.

As siderophores are commonly shared as public goods among bacterial cells and promote cooperative interactions, we next focused on their role in mediating interactions among subpopulations within PAO1 biofilms and in shaping the response to colistin treatment. We first examined the role of siderophores in promoting colistin resistance. The *pvdA* and *pchD* genes were knocked out individually to construct Δ*pvdA* and Δ*pchD* mutants. The *pvdA* and *pchE* genes were knocked out simultaneously to construct Δ*pvdA*Δ*pchE*. The effects of single and double siderophore biosynthesis loci deletions on colistin resistance were indicated by minimum inhibitory concentration (MIC). When cultivated in minimal medium with colistin, Δ*pvdA* mutant strain displayed 2-fold higher MIC than the wild-type PAO1 strain (Fig. 4A). This increase in resistance may result from the alleviation of metabolic burden associated with PVD biosynthesis (16). The Δ*pchD* strain displayed the same MIC as the wild-type strain (Fig. 4A), suggesting that the MIC remains unchanged when PVD synthesis occurs exclusively. However, the Δ*pvdA*Δ*pchE* mutant exhibited a 4-fold lower MIC than the wild-type strain (Fig. 4A), indicating the essential role of iron acquisition in colistin resistance.

To confirm that the observed decrease in MIC in Δ*pvdA*Δ*pchE* indeed resulted from the deletion of these two genes, *pvdA* and *pchE* were cloned into the plasmid pME6032 to generate the complemented strains Δ*pvdA*Δ*pchE*::pME6032-*pvdA* and Δ*pvdA*Δ*pchE*::pME6032-*pchE*. MIC tests indicated successful restoration of the phenotype of the Δ*pvdA*Δ*pchE* mutant by introducing *pvdA* or *pchE* (Fig. 4B). To confirm the role of iron in promoting colistin resistance, the iron chelator 2,2'-bipyridine was added to the medium, which caused a dose-dependent decrease in MIC values for all strains (Fig. 4C), indicating that iron limitation compromises colistin resistance. In contrast, the structurally related compound 4,4′-bipyridine, which lacks iron-chelating activity, had no effect on MIC at the same concentrations (Fig. S6). These results indicated the role of siderophores in mediating colistin resistance through iron acquisition.

**Fig 5:**
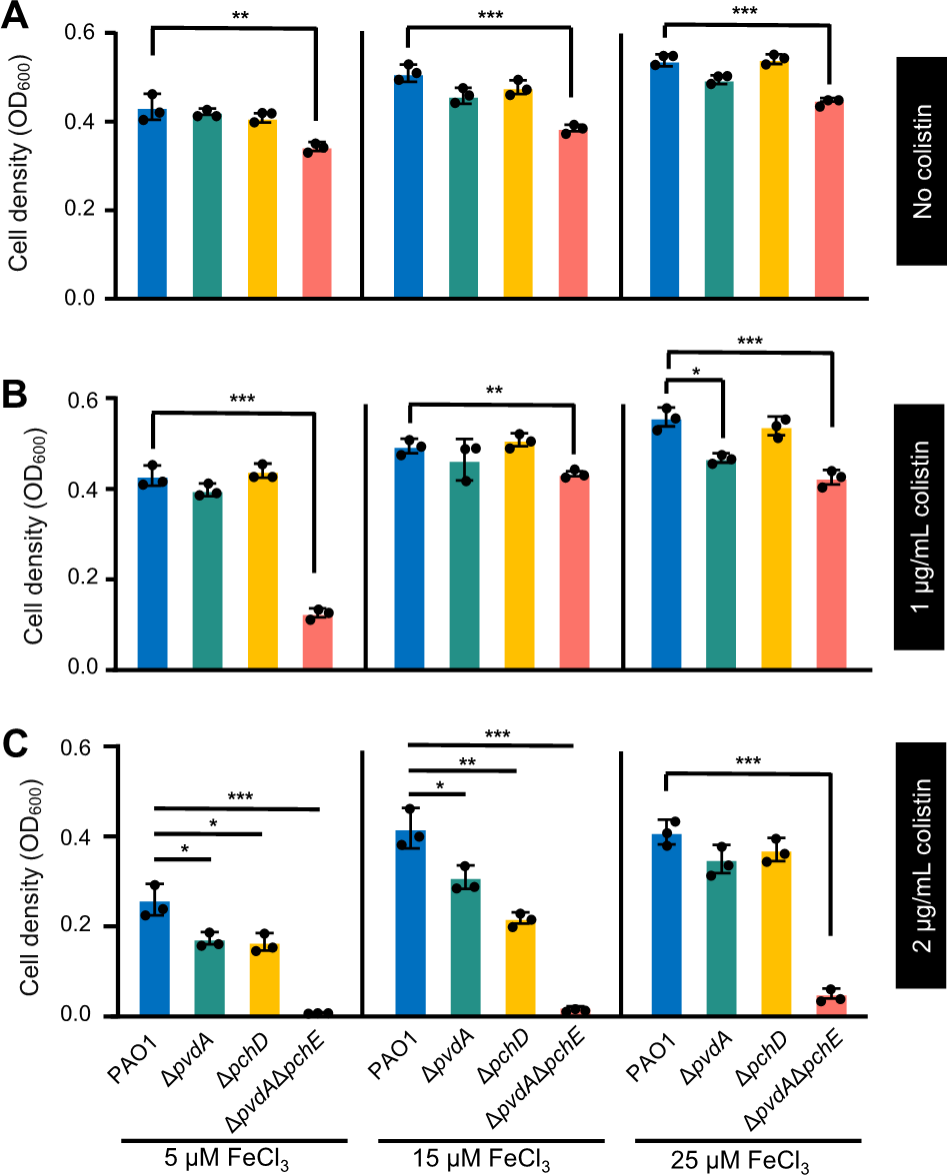
Growth of PAO1 wild-type and mutant strains under combined treatments of Fe³^+^ and colistin. (**A**) No colistin; (**B**) 1 µg/mL colistin; (**C**) 2 µg/mL colistin. All assays were performed in minimal media supplemented with the indicated concentrations of Fe³^+^ and colistin. Data represent three biological replicates, with the black dots indicating individual data points. Error bars indicate standard error based on three biological replicates. Statistical significance was determined by an unpaired two-tailed Student’s *t*-test, with *P value < 0.05, **P value < 0.01, and ***P value < 0.001.

Since iron is an essential nutrient for bacteria, the observed colistin resistance may be mediated by growth promotion. To differentiate the roles of siderophores in promoting growth versus conferring colistin resistance, we assessed the growth of the four strains under varying Fe³^+^ concentrations, both with and without different levels of colistin. In the absence of colistin, all four strains reached similar bacterial cell densities, with fold changes less than 1.3, although statistically significant differences were observed (Fig. 5A). When cultured with 1 µg/mL colistin, Δ*pvdA*Δ*pchE* exhibited a much lower cell density compared to the other three strains in the presence of 5 µM Fe³^+^, with fold changes exceeding 3 (Fig. 5B). However, supplementation with 15 µM or 25 µM Fe³^+^ restored the cell density of Δ*pvdA*Δ*pchE* (Fig. 5B). Under 2 µg/mL colistin, Δ*pvdA*Δ*pchE* again showed significantly lower cell density than the other strains, regardless of Fe³^+^ supplementation at 5 µM, 15 µM, or 25 µM (Fig. 5C). These findings demonstrate that siderophores not only facilitate bacterial growth but also contribute directly to colistin resistance.

### Uptake of exogenous siderophores promotes colistin resistance in siderophore non-producing cells

**Fig 6:**
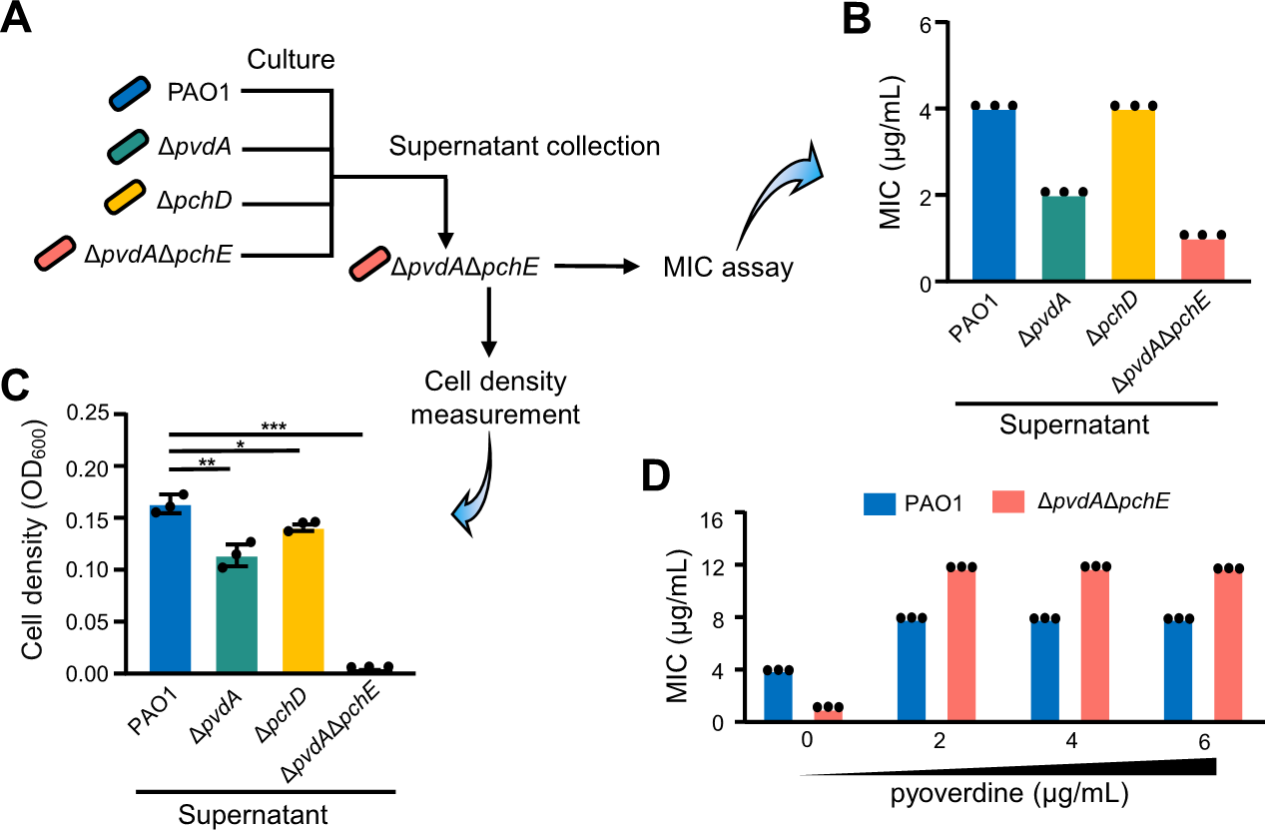
Uptake of exogenous siderophores promotes resistance in siderophore non-producing cells. (**A**) Workflow for the supernatant supplementation experiments. Filtered culture supernatants were supplemented with 25% (vol/vol) fresh succinate minimal medium to replenish nutrients before inoculation of Δ*pvdA*Δ*pchE*. (**B**) Effect of culture supernatants from indicated strains on colistin MICs for Δ*pvdA*Δ*pchE*. Data represent three biological replicates, with the black dots indicating individual data points. (**C**) Effect of culture supernatants on Δ*pvdA*Δ*pchE* cell densities after 24 h incubation under 1 µg/mL colistin treatment. Error bars indicate standard error based on three biological replicates. Significance was examined using Student’s *t*-test: *P value < 0.05, **P value < 0.01, ***P value < 0.001. (**D**) Effect of purified PVD on colistin MICs for Δ*pvdA*Δ*pchE*. Data represent three biological replicates, with the black dots indicating individual data points.

Given the specific expression of siderophore biosynthesis genes in a few subpopulations, we expected the sharing of these molecules across different subpopulations. While it is different to examine *in situ* sharing of siderophores between different subpopulations in the PAO1 biofilms, we examined the contribution of receiving exogenous siderophores to colistin resistance. MICs of Δ*pvdA*Δ*pchE* were examined in the presence of the supernatants of wild-type PAO1, Δ*pvdA*, Δ*pchD*, or Δ*pvdA*Δ*pchE* cultures (Fig. 6A). The four strains were grown in minimal medium before collection of supernatants, which were then added to Δ*pvdA*Δ*pchE* culture (Fig. 6A). Compared to the MIC in the presence of Δ*pvdA*Δ*pchE* supernatant, MIC in the presence of the supernatant of Δ*pchD* or wild-type strain was 4-fold higher, whereas the MIC in the presence of Δ*pvdA* supernatant was 2-fold higher (Fig. 6B). In parallel, the effects of the supernatants of wild-type strain, Δ*pvdA*, Δ*pchD*, and Δ*pvdA*Δ*pchE* cultures on cell density of Δ*pvdA*Δ*pchE* grown with colistin were measured (Fig. 6A). The Δ*pvdA* supernatant partially restored the cell growth of Δ*pvdA*Δ*pchE*, whereas the effect of Δ*pchD* supernatant was nearly equivalent to that of the wild-type strain supernatant (Fig. 6C). Furthermore, the effect of pure PVD compound was tested. In the minimal medium supplemented with 2, 4, or 6 µg/mL of pure PVD, the MIC of PAO1 increased from 4 to 8 µg/mL, whereas the MIC of Δ*pvdA*Δ*pchE* increased from 1 to 12 µg/mL (Fig. 6D). These results indicate that the uptake of exogenous siderophores promotes colistin resistance, with a markedly stronger effect in siderophore-deficient cells, highlighting the ecological significance of siderophore sharing among distinct subpopulations of the PAO1 strain.

### Siderophores are involved in biofilm-specific resistance and tolerance to colistin

**Fig 7:**
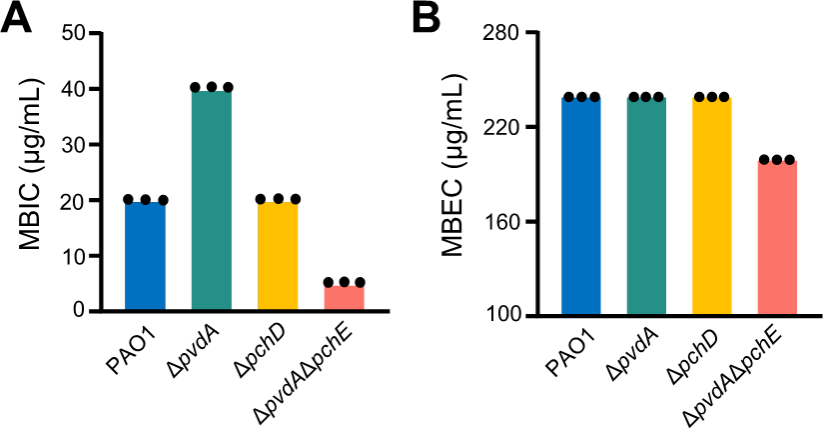
Siderophores protect PAO1 against colistin through biofilm-specific ways. (**A**) Colistin MBICs against wild-type PAO1 and mutants, determined based on OD₆₀₀ measurement of bacterial regrowth from biofilm cells after colistin treatment. (**B**) Colistin MBECs against wild-type PAO1 and mutants. Data represent three biological replicates, with the black dots indicating individual data points.

Above results demonstrated that siderophores can be shared among different subpopulations in PAO1 biofilm and this directly promotes colistin resistance, which is defined as the ability of bacteria to survive and proliferate in the presence of colistin. However, it is possible that siderophores protect PAO1 through biofilm-specific ways of antibiotic resistance or tolerance, such as enhanced biofilm forming ability and stability. To examine this notion, minimum biofilm inhibitory concentration (MBIC), which is the lowest concentration of the antimicrobial agent to inhibit the initial formation of biofilm and reflects biofilm-specific resistance to colistin, was measured for the four strains. Biofilms of the four strains were formed in a condition without colistin, and then transferred to new media with different concentrations of colistin to measure MBIC. The MBIC of Δ*pvdA*Δ*pchE* was more than 4-fold lower than that of the wild-type strain (Fig. 7A). Moreover, minimum biofilm eradication concentration (MBEC) was assessed, representing the minimal antimicrobial dose needed to completely eliminate a bacterial biofilm, and reflecting the tolerance of the PAO1 biofilms to colistin. The Δ*pvdA*Δ*pchE* mutant demonstrated the lowest MBEC among all strains tested (Fig. 7B). These results demonstrate that in addition to conferring resistance, siderophores can protect the biofilm population by promoting biofilm-relevant tolerance to colistin.

## DISCUSSION

Through the integration of single-cell transcriptomics, genetic manipulation, and physiological experiments, this study reveals social behaviors that protect clonal *P. aeruginosa* PAO1 biofilms against colistin. The core finding is that the spontaneous emergence of siderophore producer and non-producer subpopulations, along with their intercellular interaction mediated by siderophore sharing, constitutes a strategy for colistin protection.

### Social role differentiation happens within the clonal PAO1 biofilm population

For a long time, models describing bacterial public goods sharing have primarily centered on contexts of genetic diversity, including interactions between different strains, different species, or wild-type strains and mutants (17, 18). A fundamental, unresolved question has been as follows: can the differentiation of social roles still occur in a clonal population lacking genetic variation? Our research provides a clear affirmative answer to this question. scRNA-seq data clearly reveal that untreated PAO1 biofilms already possess transcriptional heterogeneity (Fig. 1), and colistin treatment significantly reshapes the subpopulation structure, specifically inducing high expression of siderophore (PVD and PCH) biosynthesis genes in subpopulation 11 (Fig. 2). Concurrently, the receptor genes for these siderophores (e.g., *fpvA* and *fptA*) are ubiquitously expressed across all subpopulations (Fig. S5). This pattern of "production specialization" and "reception universalization" empirically validates the prediction: within a group, some individuals bear the cost of producing the public good, while all individuals can benefit from it.

FISH experiments confirmed the heterogeneity of siderophore biosynthesis genes (Fig. 3), consistent with the scRNA-seq results. Additionally, these genes exhibited spatially restricted expression, forming several hotspots within the biofilm (Fig. 3). This finding supports the potential role of the biofilm microenvironment in shaping gene expression differentiation, aligning with previous findings that highlight the emergence of heterogeneous microenvironments in biofilms (19). The high expression of siderophore biosynthesis genes in subpopulation 11 may be attributed to the heterogeneous microenvironment within the biofilm. Gradients of nutrients, iron availability, and antibiotic penetration create distinct localized niches, causing only a subset of cells to experience stronger iron limitation or stress signals, thereby inducing the expression of siderophore biosynthesis genes. While the present study focused on how heterogeneous siderophore production contributes to antibiotic resistance and tolerance, less attention was paid to understanding the mechanistic origins of the heterogeneity in siderophore biosynthesis genes. These aspects warrant further investigation in future work.

### Siderophore sharing protects the PAO1 biofilms through multifaceted function

Our functional experiments demonstrate that siderophore sharing among the aforementioned cell subpopulations is not a coincidental phenomenon but is directly linked to the survival benefits of the community. Knockout of the dual siderophore system (Δ*pvdA*Δ*pchE*) led to a drastic decrease in the strain’s resistance to colistin, manifested as a 4-fold reduction in MIC, and showed significant defects in both biofilm formation and eradication assays (Fig. 4). These results underscore the essential role of siderophores in coping with colistin stress, consistent with previous findings on the involvement of siderophores in resistance or tolerance to other antibiotics, such as the siderophore-cephalosporin conjugate cefiderocol (20).

Under higher concentrations of colistin, the addition of exogenous Fe³^+^ failed to reduce the divergence in cell density between the wild-type PAO1 and the Δ*pvdA*Δ*pchE* mutant strain (Fig. 5). This observation suggests that the protective effect conferred by siderophores under antibiotic stress extends beyond merely supporting growth via iron acquisition. Instead, it implies that iron or siderophores may play additional roles in coping with colistin stress, such as maintaining the transmembrane proton motive force or modulating cellular oxidative stress, as demonstrated in previous studies (21–23).

Moreover, the resistance of the Δ*pvdA*Δ*pchE* mutant was partially or fully restored upon receiving supernatants from wild-type, Δ*pchD* (PVD-only producer), or Δ*pvdA* (PCH-only producer) strains, as well as upon the addition of purified PVD (Fig. 6). These findings confirm that siderophores secreted into the environment can function as effective public goods, rescuing cells incapable of producing them. This phenomenon mirrors the siderophore-mediated cross-species cooperation observed in polymicrobial communities (2, 3) and highlights the ecological significance of siderophore sharing as a mechanism for collective survival under antibiotic pressure. This also explains why it is not necessary for all subpopulations in the PAO1 biofilm to biosynthesize siderophores.

Furthermore, we provide evidence that siderophores protect PAO1 through biofilm-specific mechanisms, as reflected by the results of MBIC (which captures biofilm-specific resistance by measuring biofilm-forming ability under antibiotic exposure) and MBEC (which captures biofilm-specific tolerance by measuring biofilm stability under antibiotic treatment) (Fig. 7). To our knowledge, this is the first evidence linking higher MBIC or MBEC of colistin to bacterial siderophore production. Notably, the MBIC results carry two important implications: first, the MBIC of Δ*pvdA*Δ*pchE* was substantially lower than that of the other strains, confirming that siderophores protect PAO1 by promoting biofilm formation, which plays a role that is relevant not only during initial establishment but also during biofilm maturation (24); second, the MBIC of Δ*pvdA* was two-fold higher than that of the wild-type strain, suggesting that the metabolic burden of PVD biosynthesis partially compromises colistin resistance, consistent with the high metabolic cost associated with PVD production (25). The MBEC results further support the multifaceted protection conferred by siderophores against colistin. Collectively, genetic manipulation and physiological experiments demonstrate the role of siderophores in protecting PAO1 biofilms and elucidate the ecological significance of siderophore sharing among cells with differential production capacities.

### Conclusions and limitations

To conclude, by integrating cutting-edge single-cell technologies with classical microbial genetics, this study reveals how the social roles of siderophores emerge within clonal populations. Under antibiotic pressure, the *P. aeruginosa* PAO1 biofilms spontaneously form intercellular interactions through functional heterogeneity and establish a cooperative network using siderophores as important public goods. This discovery deepens our understanding of the complexity of bacterial behaviors and highlights promising therapeutic targets for combating biofilm-associated infections.

Finally, several limitations of the present study should be acknowledged. First, it is worth noting that the concentration of colistin used to treat PAO1 biofilms in this study was 40 μg/mL, which is considerably higher than the concentrations typically employed in clinical settings. This is primarily because the MBEC of colistin against PAO1 biofilms was found to be as high as 240 μg/mL (Fig. 7). In fact, several studies have suggested that none of the currently used oral or intravenous antibiotics in clinical settings are truly effective at eradicating bacterial biofilms (26, 27). It is also worth noting that in the double mutant strain (Δ*pvdA*Δ*pchE*), the gene *pchE* rather than *pchD* was disrupted, and this strain was compared to the single mutant Δ*pchE*. Given that both *pchD* and *pchE* (both involved in the PCH backbone biosynthesis from salicylate and l-cysteine) are essential for pyochelin biosynthesis (28), this choice is unlikely to significantly affect the conclusions of our study. In addition, due to the high cost of scRNA-seq, only a single replicate was used. However, our samples include two time points following colistin treatment, both of which suggest a consistent functional specification related to siderophores, which were further validated by FISH. Therefore, this limitation is unlikely to significantly affect the conclusions of our study.

## MATERIALS AND METHODS

### Bacterial strains and antibiotics

*P. aeruginosa* PAO1, a widely used biofilm model strain, was employed for biofilm study. Bacterial strains and plasmids used in this study are listed in Table S3. *P. aeruginosa* PAO1 strain was used as the parent of all derivatives used. PAO1-derived strains were cultured at 37°C in either LB medium or succinate minimal medium. The composition of the succinate minimal medium (in 1 L) was as follows: K_2_HPO_4_·3H_2_O: 6.0 g; KH_2_PO_4_: 3.0 g; (NH_4_)_2_SO_4_: 1.0 g; MgSO_4_·7H_2_O: 0.2 g; and succinic acid: 4.0 g. The pH of the medium was adjusted to 7.0 by adding NaOH. *E. coli* strains were cultured at 37°C in either LB broth or agar medium. Colistin used in this study was colistin sulfate. The stock solution of colistin (10 mg/mL) was prepared in sterile water and stored at −20°C. Stock solutions of other antibiotics were prepared as follows: kanamycin (30 mg/mL), gentamicin (10 mg/mL), chloramphenicol (30 mg/mL), and tetracycline (20 mg/mL).

### Colistin treatment

The overnight-cultured PAO1 was diluted (1:100) using fresh LB medium. Then, 2 mL of bacterial suspension was inoculated into six-well polystyrene culture plates and incubated statically at 37°C for 24 h to form visible biofilms. After biofilm formation, supernatant was discarded, and the wells were washed with 1 mL LB to remove planktonic cells. Subsequently, 2 mL of LB medium containing 40 μg/mL of colistin was added slowly to prevent biofilm destruction. In the control group, 2 mL of sterile LB medium was used. The plates were incubated at 37°C for 24 h or 36 h before sampling. After incubation, supernatants were discarded from the wells, and the biofilms were washed twice with PBS buffer to completely remove the planktonic cells and culture media.

### scRNA-seq and analysis

PAO1 biofilms, with and without colistin treatment for 24 h and 36 h, were sampled for scRNA-seq. The biofilm-derived bacterial cells were resuspended in 1 mL of 4% paraformaldehyde and fixed overnight with shaking at 4 °C. The cells were then washed and permeabilized with PBST (PBS containing 0.05% Tween 20). The cells were incubated with lysozyme at 37°C for 15 min for the digestion of the cell wall. After lysozyme treatment, bacterial cells were immediately washed and resuspended in PBS containing 0.1% RNase inhibitor. scRNA-seq library was prepared using VITAPilote kit (M20 Genomics, R20114124) according to the recommended protocol (29). Using the same protocol, total bacterial RNAs were reverse-transcribed into cDNA *in situ* employing random primer. The resulting cDNA fragment was added with adaptor. Subsequently, single-cell isolation was performed on VITACruiser Single-Cell Preparation System (M20 Genomics, Hangzhou, China). The bacterial cells were encapsulated in droplets along with magnetic beads containing cell-specific barcodes, UMIs, and DNA extension reaction mix. During the extension reaction, capture adaptors hybridized with the oligo sequences on magnetic beads. Through the extension reaction, single-cell tags were added to cDNA strands. After the extension reaction, the droplets were broken and the aqueous phase containing cDNA was purified using magnetic beads. The products were amplified by PCR and purified again using magnetic beads. Finally, the qualified libraries were sequenced on a NovaSeq 6000 System (Illumina) with paired-end reads of 150 bp.

All bioinformatic analyses were conducted on a local Linux platform, following the steps described in previous studies (30, 31). Raw sequencing data were trimmed using VITAseer (version 1.0), removing primer sequences and trimming the extra bases generated by the dA-tailing step. For each Read1, cell-specific barcode (20 nts) and UMI (8 nts) were extracted. Sequenced barcodes with a Hamming distance of 2 nt or less from an accepted barcode were uniquely assigned and merged. Read2 was mapped to the genome of PAO1 (GenBank: GCF_000006765.1) using the STARsolo module of STAR (version 2.7.10b) (32). Only uniquely mapped reads in each library were used to prepare a gene expression matrix for downstream analysis. The gene expression matrix of each library was further analyzed following the standard workflow of Seurat (version 5.0.1) (33). Cells with fewer than 10 detected genes or an abnormally high number of genes were filtered out. The “merge” function was used to merge the data frames of different samples. The “NormalizeData” function (scale.factor = 1e^4^) was applied to normalize each cell based on the total count across all genes. “FindVariableFeatures” was used for the identification of highly variable features, and “ScaleData” was applied to scale each gene to unit variance and zero mean. Then, principal component analysis (PCA) was performed using “RunPCA,” and “ElbowPlot” was employed to quickly determine the number of PCs. “IntegrateLayers” with the CCA method was used for the integration of different data sets and elimination of batch effects. A K-nearest neighbor graph was constructed using “FindNeighbors” function, based on the Euclidean distance in PCA space. The edge weights were refined using Jaccard similarity, using the previously determined number of PCs as input. The “FindClusters” function was used for clustering analysis, with a resolution of 0.7. “RunTSNE” was employed for non-linear dimensionality reduction.

To identify marker genes for each cluster, the “FindAllMarkers” function was applied, and only upregulated genes with a log₂ FoldChange > 0.25, expressed in at least 25% of cells in either cluster, and with a *P* value < 0.05 (Wilcoxon rank-sum test) were retained. Finally, the “FindMarkers” function was used to analyze differential gene expression between treatment and control groups, and only upregulated genes with a log₂ FoldChange > 0.1, expressed in at least 25% of cells in either group, and with a *P* value < 0.05 (Wilcoxon rank-sum test) were retained.

### Fluorescence *in situ* hybridization (FISH)

FISH probe was designed to target the mRNA of the *pvdA* gene. A 30 bp oligonucleotide probe was designed using Oligo 7 to achieve 100% complementarity with the mRNA sequence of the target gene. The probe was labeled with fluorochrome Cy3 at the 5′ end (excitation wavelength: 548 nm; emission wavelength: 561 nm). The sequences of the *pvdA*-Cy3 primer and other probe primers are listed in Table S4. The hybridization steps were as follows: PAO1 biofilms treated with 40 μg/mL of colistin for 24 h were prepared as described earlier (the control group was cultured in LB medium). The experiment was conducted in six-well polystyrene culture plates, placing the coverslips in the wells in advance. The biofilms were washed twice with PBST buffer to remove planktonic cells and residual culture medium. Then, 2 mL of 4% paraformaldehyde solution was added to each well to fix the biofilms for 13 h at 4°C. After removing the fixation solution, biofilms were washed again with PBST and then incubated with 2 mL of PBST at room temperature for 15-30 min to make the biofilms permeable. After permeabilization, the buffer was removed, the biofilms were covered with hybridization mix containing 100 ng/mL of *pvdA*-Cy3 probe (0.9 M NaCl, 0.02 M Tris-HCl, 0.01% SDS, and 20% formamide), and left in the dark at 46°C for 5–8 h. After hybridization, the biofilms were incubated with 2 mL of wash buffer (0.1 M NaCl, 0.02 M Tris-HCl, 0.01% SDS, and 5 mM EDTA) at 48°C for 15 min. Afterward, biofilms were rinsed with PBS to remove the wash buffer, and then, 1 mL of 1 μg/mL of 4′,6-diamidino-2-phenylindole (DAPI, excitation wavelength: 353 nm, emission wavelength: 465 nm) was added to biofilms. After incubation at room temperature in the dark for 15 min, biofilms were washed twice with PBS. The coverslips containing biofilms were removed and placed on a glass slide with a drop of antifade mounting medium. The obtained biofilm samples were observed using a confocal laser scanning microscopy (CLSM, Zeiss LSM900) at the Fang Zongxi Center, Ocean University of China (Qingdao, China).

### Construction of gene knock-out mutant

Gene knock-out mutant of PAO1 was constructed using homologous recombination mediated by pK18*mobSacB*. To construct the knock-out plasmid for deletion of *pvdA* gene, the 713 bp upstream fragment and 729 bp downstream fragment flanking *pvdA* were amplified using *pvdA*-up-F/*pvdA*-up-R and *pvdA*-low-F/*pvdA*-low-R primer pairs. The upstream and downstream PCR fragments were ligated by overlapping PCR, and the resulting PCR products were inserted into the EcoRI/XbaI sites of suicide vector pK18*mobSacB*. The gentamicin resistance cassette amplified from p34s-Gm was subsequently inserted into the same HindIII site to obtain the knock-out plasmid pK18-Δ*pvdA*-Gm. The pK18-Δ*pvdA*-Gm plasmid was transformed into *E. coli* S17-1 through heat shock. The donor strain S17-1 (pK18-Δ*pvdA*-Gm) was obtained through selection on LB agar containing 30 μg/mL of kanamycin and 10 μg/mL of gentamicin. Subsequently, PAO1 was used as the recipient strain, and the mixtures of donor and recipient (1:3) were incubated on LB agar plates at 37°C for 48 h. To select PAO1 transconjugants, cells were resuspended in PBS and plated onto LB agar plates containing 200 μg/mL of gentamicin and 30 μg/mL of chloramphenicol. Selected transconjugants were then inoculated into liquid LB medium and cultured overnight at 37°C. Obtained bacterial suspension was serially diluted and plated onto LB agar plates containing 12% sucrose. The *pvdA* gene deletion mutant Δ*pvdA* was obtained through PCR and antibiotic resistance selection. Successful construction of mutant was further confirmed through Sanger sequencing. The mutant strains Δ*pchD* and Δ*pvdA*Δ*pchE* were constructed using the same method. All primers used for homologous recombination are listed in Table S4.

### Construction of complementary strains

To prepare the complementary strain of Δ*pvdA*Δ*pchE* mutant, PCR-amplified *pvdA* was cloned into SacI and XhoI sites of plasmid pME6032, generating pME6032-*pvdA*. This recombinant plasmid was then transformed into Δ*pvdA*Δ*pchE* by electroporation and selected on the LB agar plates containing 30 μg/mL kanamycin and 150 μg/mL tetracycline. Other complementary strains were constructed using the same method. During the antibiotic resistance experiments, 1 mM of IPTG was added into the cultures to induce gene expression. All primers used for construction of the complemented strains are listed in Table S4.

### MIC determination

The MIC of colistin was determined by standard serial 2-fold microdilution method using succinate minimal medium (composition provided above). To construct iron-limited conditions, 2,2′-bipyridine (an iron chelator that preferentially binds Fe²^+^) was added at a series of concentrations (i.e., 100, 250, and 500 μM). Likewise, the effect of 4,4′-bipyridine (a structurally related compound lacking iron-chelating activity) on colistin MICs was assessed. The wild-type and mutant strains of PAO1 were cultured overnight under the corresponding conditions. After overnight incubation, bacterial suspensions were diluted to an approximate cell density of 10⁵ CFUs/mL using the respective media. Subsequently, 20 μL of colistin solution, with a concentration of 160 μg/mL, was serially diluted 2-fold in a 96-well plate. Afterward, 180 μL of the diluted bacterial suspension was added to each well. The negative control consisted of 20 μL of water. The 96-well plate was incubated at 37°C for 24 h. In the presence of iron chelator 2,2′-bipyridine, the incubation time was extended to 48 h. After incubation, OD₆₀₀ values of bacterial cultures were measured. The MIC was defined as the lowest concentration of colistin required for total inhibition of bacterial growth. To investigate the influence of supernatants of different bacterial cultures on MIC, the wild-type and mutant strains of PAO1 cultured overnight were inoculated into 10 mL of fresh minimal medium at 1% inoculum concentration, and then incubated for 48 h at 37°C with constant shaking. The obtained bacterial suspension was centrifuged at 6,000 rpm for 8 min, and then the supernatant was collected and filtered through a 0.22 μm polytetrafluoroethylene filter membrane. Subsequently, 25% fresh minimal medium was added to the filtered supernatant to replenish the nutrients, followed by the inoculation of the Δ*pvdA*Δ*pchE* culture, and the addition of a series of concentrations of colistin. After cultivation for 24 h, MIC was measured by following the steps described above.

### Minimum biofilm inhibitory concentration (MBIC) and minimum biofilm eradication concentration (MBEC) determination

Overnight cultures of PAO1 wild-type and mutant strains were grown in minimal medium and diluted to an approximate cell density of 5 × 10^5^ CFU/ml; 150 µL of bacterial dilution was inoculated into each well of a 96-well plate, covered with a lid containing pegs for the attachment of the bacteria (Biofilm Drug Killing Effect Assay Kit). The plate was incubated at 37°C for 36 h to form biofilms. The plate lids containing the pegs with the attached biofilms were removed and transferred to a new 96-well plate, in which each well was filled with a 2-fold serial dilution of colistin solution prepared in minimal medium. The plate was incubated at 37°C for 48 h. After incubation, OD₆₀₀ values of bacterial cultures were measured. The MBIC was defined as the lowest concentration of colistin required for inhibition of biofilm bacterial growth. The peg lid was then rinsed twice with PBS and transferred to a fresh 96-well plate containing LB medium. After incubation at 37°C for 24 h, the MBEC was determined as the lowest antibiotic concentration at which no visible bacterial regrowth was observed in the wells. The MBEC was defined as the minimum antibiotic concentration required to eradicate biofilm bacteria, including dormant cells.

### Statistical analysis

Differential expression analysis was performed using R (version 4.3.2) (34). For single-cell RNA-seq data, the Wilcoxon rank-sum test was applied via the “FindAllMarkers” and “FindMarkers” functions in Seurat (version 5.0.1). Genes were considered differentially expressed if they met the following criteria: log₂ FoldChange > 0.25 (for FindAllMarkers) or > 0.1 (for FindMarkers); expression in at least 25% of cells in either cluster or group; and a *P* value < 0.05. For bacterial physiological assays, differences between groups were evaluated using an unpaired two-tailed Student’s *t*-test. Significance thresholds were defined as follows: *P value < 0.05, **P value < 0.01, and ***P value < 0.001. All experiments were conducted with at least three independent biological replicates.

## Data Availability

The raw sequence data reported in this paper have been deposited in the Genome Sequence Archive in National Genomics Data Center, China National Center for Bioinformation/Beijing Institute of Genomics, Chinese Academy of Sciences (https://ngdc.cncb.ac.cn/gsa/), and are publicly accessible under accession no. CRA023293, CRA023279, and CRA028665.

## SUPPLEMENTAL MATERIAL

Supplemental material aem.00588-26-s0001.docx Fig. S1 to S6; Tables S1 to S4.

ASM does not own the copyrights to Supplemental Material that may be linked to, or accessed through, an article. The authors have granted ASM a non-exclusive, world-wide license to publish the Supplemental Material files. Please contact the corresponding author directly for reuse.

## References

[1] Murdoch CC, Skaar EP. 2022. Nutritional immunity: the battle for nutrient metals at the host-pathogen interface. Nat Rev Microbiol 20:657–670. doi:10.1038/s41579-022-00745-6 35641670 PMC9153222

[2] Schalk IJ. 2025. Bacterial siderophores: diversity, uptake pathways and applications. Nat Rev Microbiol 23:24–40. doi:10.1038/s41579-024-01090-6 39251840

[3] Wei Z, Gu S, Vollenweider V, Zuo Y, Li Z, Kümmerli R. 2025. Microbial siderophores for One Health. Trends Microbiol 33:1277–1285. doi:10.1016/j.tim.2025.05.002 40467390

[4] Sachdeva C, Satyamoorthy K, Murali TS. 2025. Pseudomonas aeruginosa: metabolic allies and adversaries in the world of polymicrobial infections. Crit Rev Microbiol 51:619–638. doi:10.1080/1040841X.2024.2397359 39225080

[5] Besse A, Menetrey Q, Jean-Pierre V, Huc-Brandt S, Aujoulat F, Dupont C, Chiron R, Armengaud J, Jumas-Bilak E, Molle V, et al.. 2025. Achromobacter xylosoxidans modulates Pseudomonas aeruginosa virulence through a complex multi-target competition. Sci Rep 15:23392. doi:10.1038/s41598-025-06075-w 40603430 PMC12223198

[6] Butaitė E, Baumgartner M, Wyder S, Kümmerli R. 2017. Siderophore cheating and cheating resistance shape competition for iron in soil and freshwater Pseudomonas communities. Nat Commun 8:414. doi:10.1038/s41467-017-00509-4 28871205 PMC5583256

[7] Lissens M, Joos M, Lories B, Steenackers HP. 2022. Evolution-proof inhibitors of public good cooperation: a screening strategy inspired by social evolution theory. FEMS Microbiol Rev 46:fuac019. doi:10.1093/femsre/fuac019 35675280 PMC9616471

[8] Williamson KS, Richards LA, Perez-Osorio AC, Pitts B, McInnerney K, Stewart PS, Franklin MJ. 2012. Heterogeneity in Pseudomonas aeruginosa biofilms includes expression of ribosome hibernation factors in the antibiotic-tolerant subpopulation and hypoxia-induced stress response in the metabolically active population. J Bacteriol 194:2062–2073. doi:10.1128/JB.00022-12 22343293 PMC3318454

[9] Ma P, Amemiya HM, He LL, Gandhi SJ, Nicol R, Bhattacharyya RP, Smillie CS, Hung DT. 2023. Bacterial droplet-based single-cell RNA-seq reveals antibiotic-associated heterogeneous cellular states. Cell 186:877–891. doi:10.1016/j.cell.2023.01.002 36708705 PMC10014032

[10] Korshoj LE, Kielian T. 2024. Bacterial single-cell RNA sequencing captures biofilm transcriptional heterogeneity and differential responses to immune pressure. Nat Commun 15:10184. doi:10.1038/s41467-024-54581-8 39580490 PMC11585574

[11] Yan X, Liao H, Wang C, Huang C, Zhang W, Guo C, Pu Y. 2024. An improved bacterial single-cell RNA-seq reveals biofilm heterogeneity. eLife 13:RP97543. doi:10.7554/eLife.97543 39689163 PMC11651652

[12] Meng H, Zhang T, Wang Z, Zhu Y, Yu Y, Chen H, Chen J, Wang F, Yu Y, Hua X, Wang Y. 2024. High-throughput host-microbe single-cell RNA sequencing reveals ferroptosis-associated heterogeneity during Acinetobacter baumannii infection. Angew Chem Int Ed 63:e202400538. doi:10.1002/anie.202400538 38419141

[13] Jo J, Price-Whelan A, Dietrich LEP. 2022. Gradients and consequences of heterogeneity in biofilms. Nat Rev Microbiol 20:593–607. doi:10.1038/s41579-022-00692-2 35149841 PMC9590228

[14] Krell T, Matilla MA. 2024. Pseudomonas aeruginosa. Trends Microbiol 32:216–218. doi:10.1016/j.tim.2023.11.005 38065787

[15] Mridha S, Kümmerli R. 2022. Coordination of siderophore gene expression among clonal cells of the bacterium Pseudomonas aeruginosa. Commun Biol 5:545. doi:10.1038/s42003-022-03493-8 35668142 PMC9170778

[16] Dumas Z, Ross-Gillespie A, Kümmerli R. 2013. Switching between apparently redundant iron-uptake mechanisms benefits bacteria in changeable environments. Proc Biol Sci 280:20131055. doi:10.1098/rspb.2013.1055 23760867 PMC3712426

[17] Özkaya Ö, Xavier KB, Dionisio F, Balbontín R. 2017. Maintenance of microbial cooperation mediated by public goods in single-and multiple-trait scenarios. J Bacteriol 199:10–1128. doi:10.1128/JB.00297-17 PMC5648865 28847922

[18] Smith P, Schuster M. 2019. Public goods and cheating in microbes. Curr Biol 29:R442–R447. doi:10.1016/j.cub.2019.03.001 31163154

[19] Ren Y, Wang C, Chen Z, Allan E, van der Mei HC, Busscher HJ. 2018. Emergent heterogeneous microenvironments in biofilms: substratum surface heterogeneity and bacterial adhesion force-sensing. FEMS Microbiol Rev 42:259–272. doi:10.1093/femsre/fuy001 29325130

[20] Galdino ACM, Vaillancourt M, Celedonio D, Huse K, Doi Y, Lee JS, Jorth P. 2024. Siderophores promote cooperative interspecies and intraspecies cross-protection against antibiotics in vitro. Nat Microbiol 9:631–646. doi:10.1038/s41564-024-01601-4 38409256 PMC11239084

[21] Short KA, Blakemore RP. 1986. Iron respiration-driven proton translocation in aerobic bacteria. J Bacteriol 167:729–731. doi:10.1128/jb.167.2.729-731.1986 3015890 PMC212953

[22] Cornelis P, Wei Q, Andrews SC, Vinckx T. 2011. Iron homeostasis and management of oxidative stress response in bacteria. Metallomics 3:540–549. doi:10.1039/c1mt00022e 21566833

[23] Yang B, Tong Z, Shi J, Wang Z, Liu Y. 2023. Bacterial proton motive force as an unprecedented target to control antimicrobial resistance. Med Res Rev 43:1068–1090. doi:10.1002/med.21946 36896761

[24] Liaqat I, Liaqat M, Ali S, Ali NM, Haneef U, Mirza SA, Tahir HM. 2019. Biofilm formation, maturation and prevention: a review. J Bacteriol Mycol 6:1092. doi:10.26420/JBACTERIOLMYCOL.2019.1092

[25] Anderson AJ, Kim YC. 2020. Insights into plant-beneficial traits of probiotic Pseudomonas chlororaphis isolates. J Med Microbiol 69:361–371. doi:10.1099/jmm.0.001157 32043956

[26] Lebeaux D, Ghigo JM, Beloin C. 2014. Biofilm-related infections: bridging the gap between clinical management and fundamental aspects of recalcitrance toward antibiotics. Microbiol Mol Biol Rev 78:510–543. doi:10.1128/MMBR.00013-14 25184564 PMC4187679

[27] Wu H, Moser C, Wang HZ, Høiby N, Song ZJ. 2015. Strategies for combating bacterial biofilm infections. Int J Oral Sci 7:1–7. doi:10.1038/ijos.2014.65 25504208 PMC4817533

[28] Reimmann C, Patel HM, Serino L, Barone M, Walsh CT, Haas D. 2001. Essential PchG-dependent reduction in pyochelin biosynthesis of Pseudomonas aeruginosa. J Bacteriol 183:813–820. doi:10.1128/JB.183.3.813-820.2001 11208777 PMC94946

[29] Xu Z, Wang Y, Sheng K, Rosenthal R, Liu N, Hua X, Zhang T, Chen J, Song M, Lv Y, et al.. 2023. Droplet-based high-throughput single microbe RNA sequencing by smRandom-seq. Nat Commun 14:5130. doi:10.1038/s41467-023-40137-9 37612289 PMC10447461

[30] Ding W, Wang S, Qin P, Fan S, Su X, Cai P, Lu J, Cui H, Wang M, Shu Y, et al.. 2023. Anaerobic thiosulfate oxidation by the Roseobacter group is prevalent in marine biofilms. Nat Commun 14:2033. doi:10.1038/s41467-023-37759-4 37041201 PMC10090131

[31] Fan S, Qin P, Lu J, Wang S, Zhang J, Wang Y, Cheng A, Cao Y, Ding W, Zhang W. 2024. Bioprospecting of culturable marine biofilm bacteria for novel antimicrobial peptides. iMeta 3:e244. doi:10.1002/imt2.244 39742298 PMC11683478

[32] Dobin A, Davis CA, Schlesinger F, Drenkow J, Zaleski C, Jha S, Batut P, Chaisson M, Gingeras TR. 2013. STAR: ultrafast universal RNA-seq aligner. Bioinformatics 29:15–21. doi:10.1093/bioinformatics/bts635 23104886 PMC3530905

[33] Stuart T, Butler A, Hoffman P, Hafemeister C, Papalexi E, Mauck WM III, Hao Y, Stoeckius M, Smibert P, Satija R. 2019. Comprehensive integration of single-cell data. Cell 177:1888–1902. doi:10.1016/j.cell.2019.05.031 31178118 PMC6687398

[34] Ihaka R, Gentleman R. 1996. R: a language for data analysis and graphics. J Comput Graph Stat 5:299–314. doi:10.1080/10618600.1996.10474713

