# Supplementary material for: Siderophore sharing protects clonal Pseudomonas aeruginosa biofilms from colistin
Source: Appl Environ Microbiol. 2026 Jun 17;92(7):e00588-26. doi: 10.1128/aem.00588-26 (PMC13390480; doi:10.1128/aem.00588-26)
Supplement: Supplemental material — Fig. S1 to S6; Tables S1 to S4. [file aem.00588-26-s0001.docx]

**Supporting Information**

Wang et al.,

**Siderophore sharing protects clonal *Pseudomonas aeruginosa* biofilms from colistin**

This file includes Fig. S1-S6 & Table S1-S4.


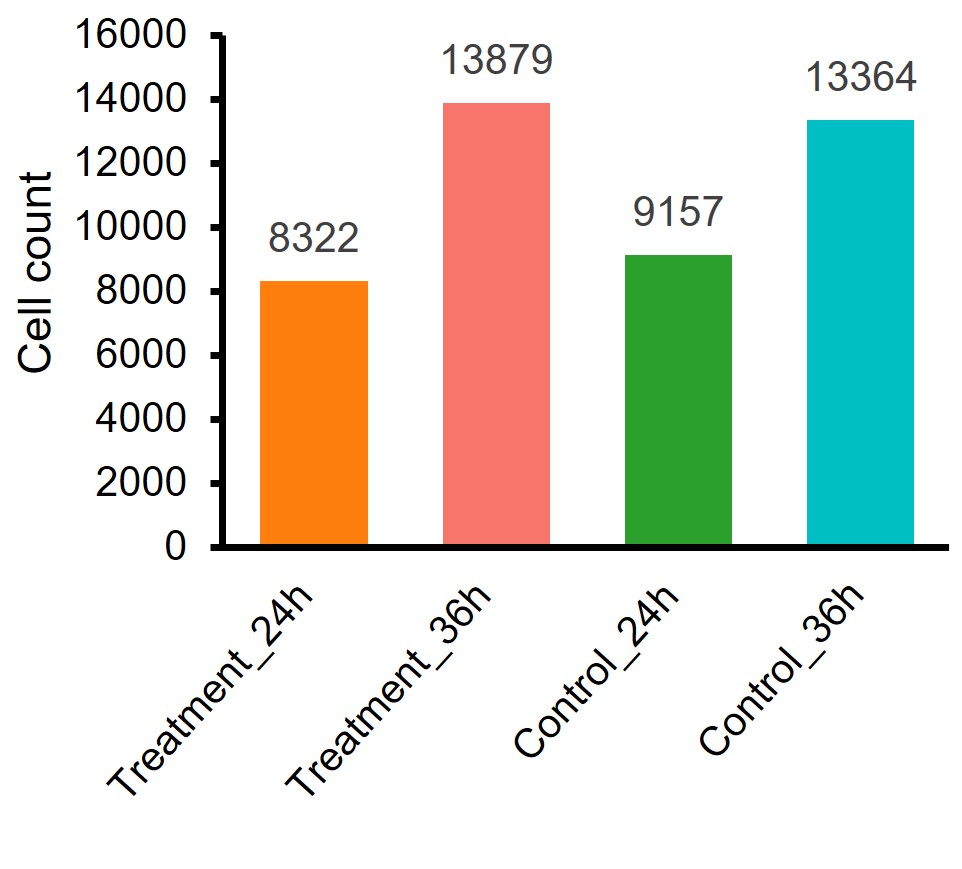


**Fig. S1** Bacterial cell count in the scRNA-seq dataset. Cell numbers were determined from quality-filtered reads.


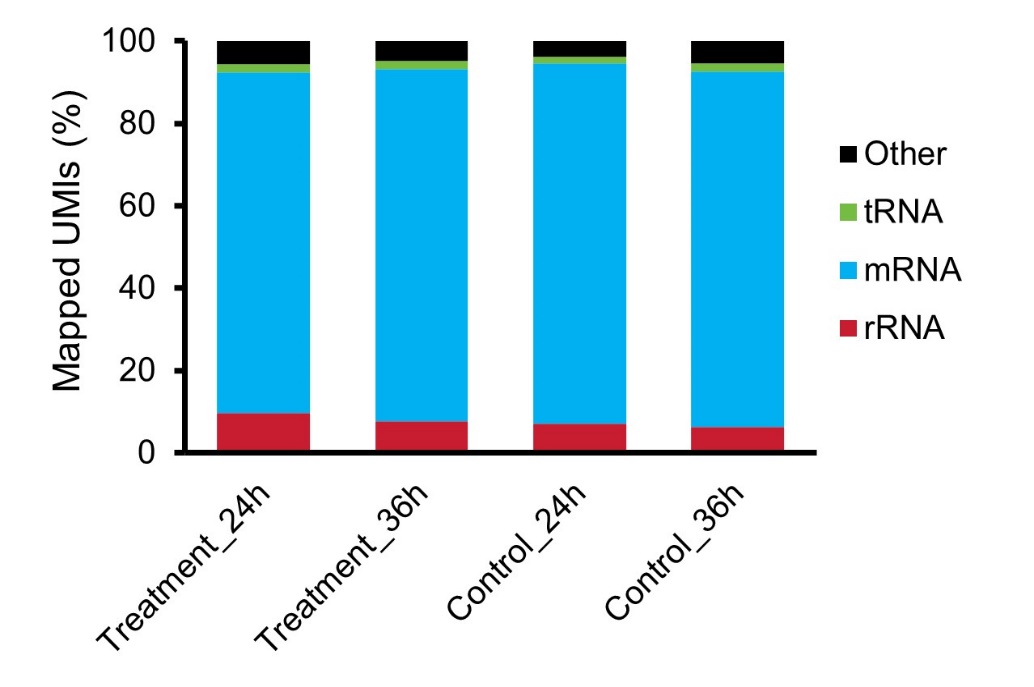


**Fig. S2** RNA type composition among unique molecular identifiers (UMIs) derived from the scRNA-seq dataset.

**
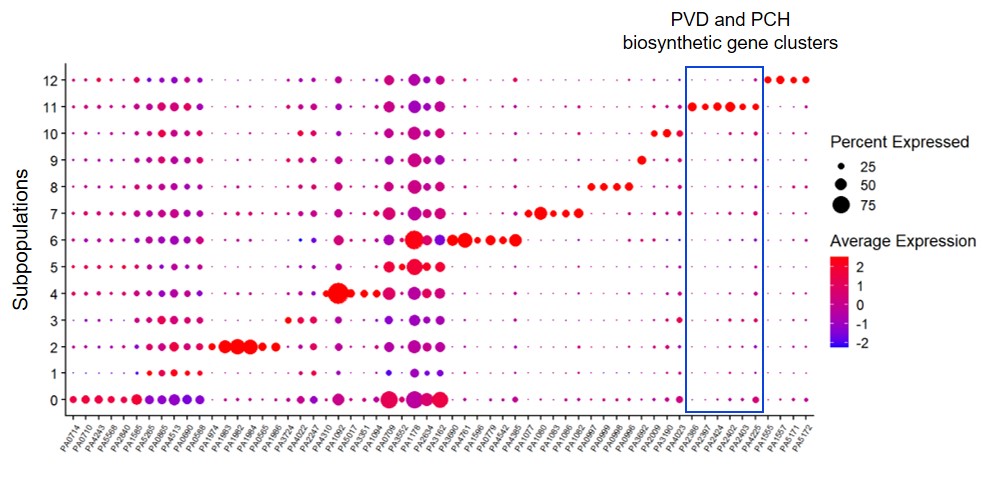
Fig. S3** Identification and visualization of specifically enriched genes across cellular subpopulations. The blue box highlights the biosynthetic gene clusters for the siderophores PVD and PCH.

**
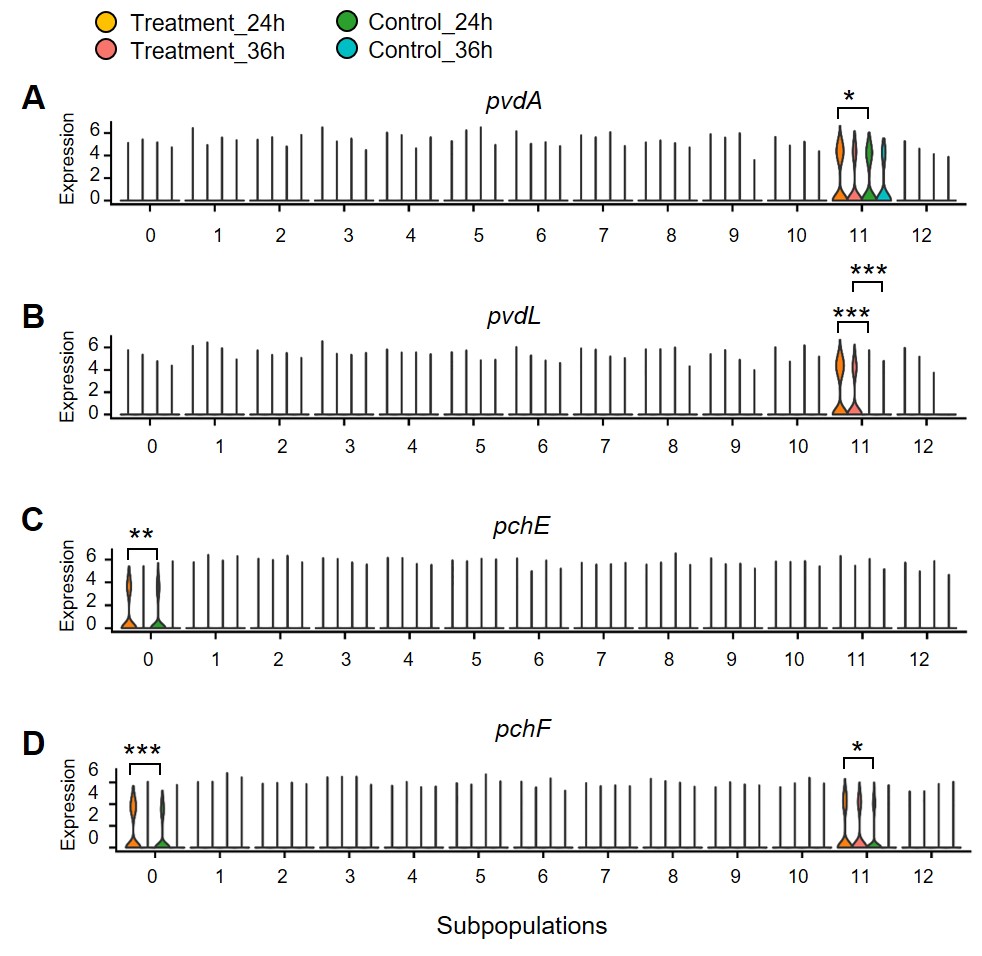
Fig. S4** Single-cell expression profiles of siderophore biosynthesis genes. Violin plots depict expression patterns of *pvdA* (**A**), *pvdL* (**B**), *pchE* (**C**), and *pchF* (**D**) across all cell clusters in 24-h and 36-h treatment/control groups. Significance markers denote differences between treatment and control groups (Wilcoxon rank-sum test: * *P* value < 0.05, ** *P* value < 0.01, *** *P* value < 0.001).

**
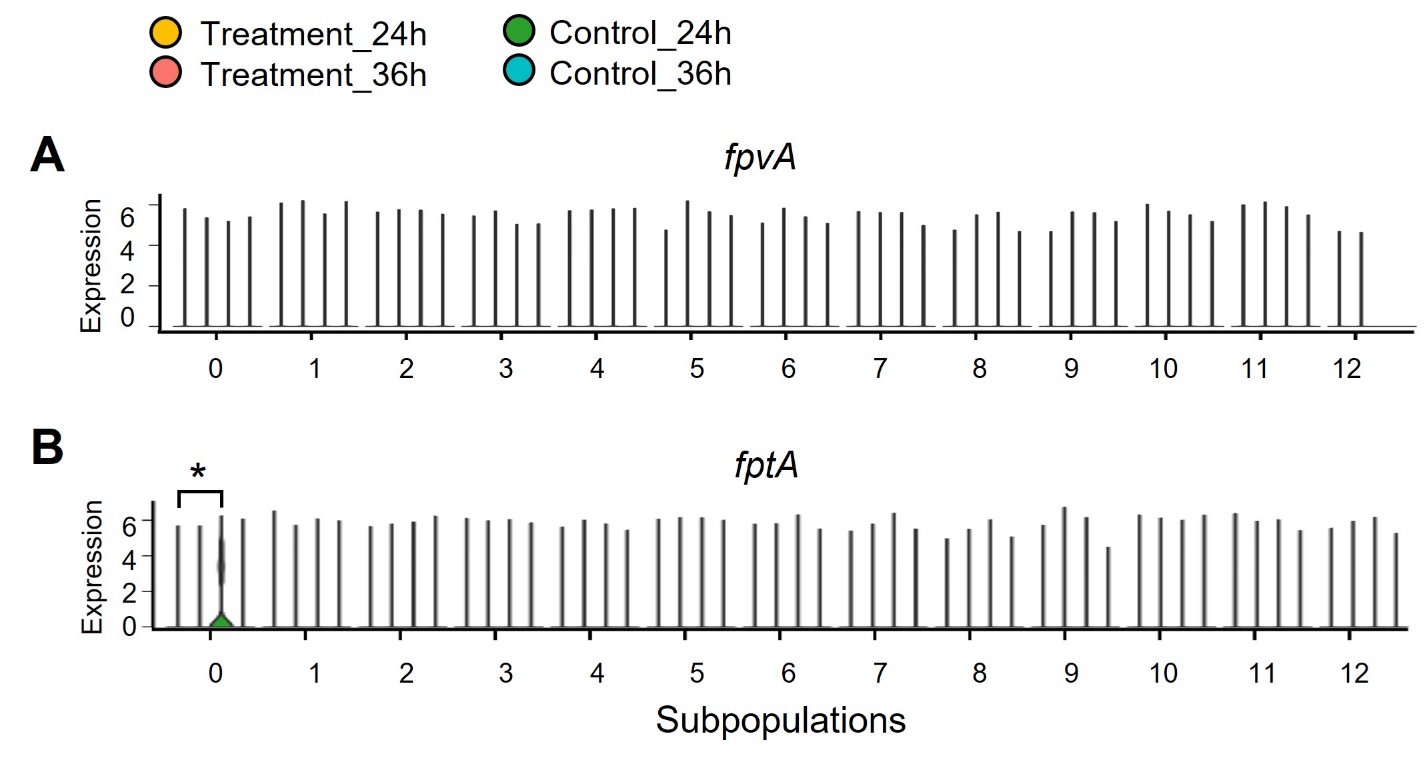
**

**Fig. S5** Single-cell expression profiles of siderophore receptor genes. Violin plots depict expression patterns of *fpvA* (encodes PVD receptor; **A**) and *fptA* (encodes PCH receptor; **B**) across all cell clusters in 24-h and 36-h treatment/control groups. Significance markers denote differences between treatment and control groups (Wilcoxon rank-sum test: * *P* value < 0.05, ** *P* value < 0.01, *** *P* value < 0.001).


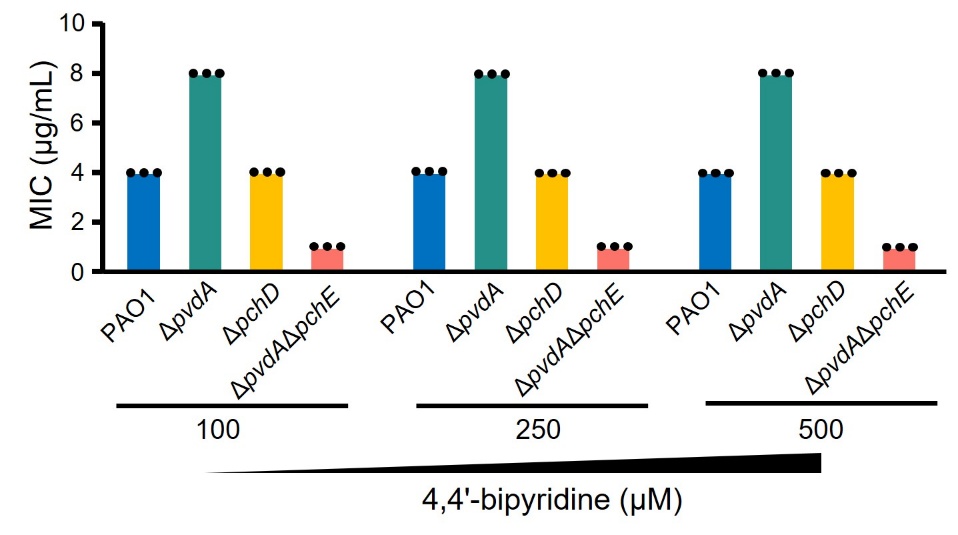


**Fig. S6** Effect of exogenous iron chelator 4,4'-bipyridine on colistin MICs. 4,4'-bipyridine is a structural analog of 2,2'-bipyridine but it does not chelate iron. Data represent three biological replicates, with the black dots indicating individual data points. Because the three values derived from the three biological replicates are identical, no error bars are shown.

**Table S1** scRNA-seq data information.

|  | **Treatment_24h** | | **Control_24h** | |
| --- | --- | --- | --- | --- |
|  | **STAR Filtering** | **Seurat  Second Filtering** | **STAR Filtering** | **Seurat  Second Filtering** |
| Number of Reads | 185724162 |  | 184453922 |  |
| Reads With Valid Barcodes | 0.99829 |  | 0.998397 |  |
| Sequencing Saturation | 0.677818 |  | 0.710856 |  |
| Q30 Bases in CB+UMI | 0.969052 |  | 0.965134 |  |
| Q30 Bases in RNA read | 0.926654 |  | 0.920918 |  |
| Reads Mapped to Genome: Unique+Multiple | 0.909599 |  | 0.909002 |  |
| Reads Mapped to Genome: Unique | 0.106433 |  | 0.146977 |  |
| Reads Mapped to GeneFull: Unique+Multiple GeneFull | NoMulti |  | NoMulti |  |
| Reads Mapped to GeneFull: Unique GeneFull | 0.081656 |  | 0.115857 |  |
| **Estimated Number of Cells** | 8322 | 8322 | 9157 | 9157 |
| Unique Reads in Cells Mapped to GeneFull | 6161743 |  | 8453508 |  |
| Fraction of Unique Reads in Cells | 0.4063 |  | 0.395572 |  |
| Mean Reads per Cell | 740 |  | 923 |  |
| Median Reads per Cell | 591 |  | 665 |  |
| UMIs in Cells | 1651221 |  | 1993018 |  |
| Mean UMI per Cell | 198 |  | 217 |  |
| **Median UMI per Cell** | 161 | 161 | 159 | 159 |
| Mean GeneFull per Cell | 143 |  | 143 |  |
| **Median GeneFull per Cell** | 120 | 120 | 111 | 111 |
| Total GeneFull Detected | 5571 |  | 5572 |  |
| **Data accession in CNCB** | CRA023293 |  | CRA023293 |  |
|  |  |  |  |  |
|  | **Treatment_36h** | | **Control_36h** | |
|  | **STAR Filtering** | **Seurat  Second Filtering** | **STAR Filtering** | **Seurat  Second Filtering** |
| Number of Reads | 203327223 |  | 174869497 |  |
| Reads With Valid Barcodes | 0.998105 |  | 0.998197 |  |
| Sequencing Saturation | 0.607696 |  | 0.632871 |  |
| Q30 Bases in CB+UMI | 0.961807 |  | 0.966756 |  |
| Q30 Bases in RNA read | 0.901906 |  | 0.915732 |  |
| Reads Mapped to Genome: Unique+Multiple | 0.888856 |  | 0.911705 |  |
| Reads Mapped to Genome: Unique | 0.130621 |  | 0.14879 |  |
| Reads Mapped to GeneFull: Unique+Multiple GeneFull | NoMulti |  | NoMulti |  |
| Reads Mapped to GeneFull: Unique GeneFull | 0.100549 |  | 0.115307 |  |
| **Estimated Number of Cells** | 13881 | 13879 | 13364 | 13364 |
| Unique Reads in Cells Mapped to GeneFull | 7966112 |  | 8712931 |  |
| Fraction of Unique Reads in Cells | 0.389651 |  | 0.432111 |  |
| Mean Reads per Cell | 573 |  | 651 |  |
| Median Reads per Cell | 471 |  | 504 |  |
| UMIs in Cells | 2725049 |  | 2799981 |  |
| Mean UMI per Cell | 196 |  | 209 |  |
| **Median UMI per Cell** | 162 | 162 | 163 | 163 |
| Mean GeneFull per Cell | 140 |  | 147 |  |
| **Median GeneFull per Cell** | 119 | 119 | 120 | 120 |
| Total GeneFull Detected | 5572 |  | 5572 |  |
| **Data accession in CNCB** | CRA023293 |  | CRA023293 |  |

**Table S2** Full list of differentially expressed genes in each subpopulation identified by scRNA-seq. Significance: Log_2_ FoldChange> 0.1, *P* value < 0.05, and expression in > 25% of cells.

| **Treatment_24h vs. Control_24h** | **Gene** | **Gene name** | **KEGG annotation** | ***P* value** | **Log2 FoldChange** | **pct.1** | **pct.2** |
| --- | --- | --- | --- | --- | --- | --- | --- |
| 0 | PA2402 | *pvdI* |  | 5.35E-36 | 1.883668046 | 0.335 | 0.128 |
| 0 | PA3724 | *lasB* | K01399 | 3.52E-34 | 1.767599349 | 0.316 | 0.116 |
| 0 | PA3049 | *rmf* | K03812 | 1.15E-17 | 1.129757007 | 0.343 | 0.191 |
| 0 | PA5429 | *aspA* | K01744 | 2.40E-13 | 0.92746932 | 0.334 | 0.203 |
| 0 | PA4225 | *pchF* | K12240 | 8.24E-09 | 0.745307567 | 0.407 | 0.306 |
| 0 | PA4525 | *pilA* | K02650 | 1.25E-07 | 0.759676872 | 0.345 | 0.254 |
| 0 | PA5446 |  |  | 2.21E-07 | 0.906088771 | 0.269 | 0.184 |
| 0 | PA0865 | *hpd* | K00457 | 1.36E-06 | 0.401479367 | 0.526 | 0.44 |
| 0 | PA4513 |  | K00380 | 1.54E-05 | 0.405364445 | 0.512 | 0.435 |
| 0 | PA0456 |  | K03704 | 2.70E-05 | 0.474178876 | 0.413 | 0.341 |
| 0 | PA1585 | *sucA* | K00164 | 4.42E-05 | 0.317131254 | 0.523 | 0.446 |
| 0 | PA4133 |  | K00404 | 7.52E-05 | 0.603172562 | 0.333 | 0.265 |
| 0 | PA0900 |  |  | 0.000240101 | 0.483457545 | 0.399 | 0.342 |
| 0 | PA4347 |  |  | 0.000284554 | 0.409701782 | 0.442 | 0.394 |
| 0 | PA0872 | *phhA* | K00500 | 0.000779216 | 0.47358507 | 0.308 | 0.253 |
| 0 | PA4747 | *secG* | K03075 | 0.003525999 | 0.477965162 | 0.369 | 0.323 |
| 0 | PA4226 | *pchE* | K12239 | 0.004996938 | 0.298682281 | 0.332 | 0.282 |
| 0 | PA0690 |  |  | 0.006848226 | 0.475734019 | 0.441 | 0.409 |
| 0 | PA4922 | *azu* |  | 0.009733145 | 0.367047424 | 0.34 | 0.296 |
| 0 | PA5187 |  | K20035 | 0.011988813 | 0.393125237 | 0.3 | 0.258 |
| 0 | PA5349 |  | K05297 | 0.01223794 | 0.416109562 | 0.334 | 0.302 |
| 0 | PA4260 | *rplB* | K02886 | 0.024633434 | 0.290055701 | 0.37 | 0.338 |
| 0 | PA5248 |  | K07243 | 0.02972979 | 0.377099 | 0.251 | 0.217 |
| 0 | PA0139 | *ahpC* | K24119 | 0.037586755 | 0.504987436 | 0.265 | 0.237 |
| 0 | PA3622 | *rpoS* | K03087 | 0.038360601 | 0.302418623 | 0.285 | 0.253 |
| 0 | PA5078 |  | K03670 | 0.038849818 | 0.395493337 | 0.314 | 0.288 |
| 1 | PA0865 | *hpd* | K00457 | 1.14E-29 | 0.998587121 | 0.381 | 0.194 |
| 1 | PA3049 | *rmf* | K03812 | 2.94E-25 | 0.783022451 | 0.299 | 0.138 |
| 1 | PA4513 |  | K00380 | 6.45E-13 | 0.500583592 | 0.363 | 0.236 |
| 1 | PA4347 |  |  | 1.10E-12 | 0.590814005 | 0.31 | 0.189 |
| 1 | PA2247 | *bkdA1* | K00166 | 5.94E-08 | 0.438275685 | 0.262 | 0.178 |
| 1 | PA5265 |  |  | 8.13E-07 | 0.279146503 | 0.26 | 0.178 |
| 1 | PA0690 |  |  | 0.000203936 | 0.20167753 | 0.263 | 0.195 |
| 2 | PA3049 | *rmf* | K03812 | 6.70E-14 | 1.35055141 | 0.377 | 0.177 |
| 2 | PA2402 | *pvdI* |  | 1.80E-12 | 1.824399126 | 0.258 | 0.094 |
| 2 | PA0872 | *phhA* | K00500 | 5.44E-07 | 1.124370972 | 0.281 | 0.156 |
| 2 | PA4347 |  |  | 9.99E-06 | 0.628232265 | 0.383 | 0.262 |
| 2 | PA0865 | *hpd* | K00457 | 0.000222088 | 0.385554791 | 0.488 | 0.362 |
| 2 | PA0456 |  | K03704 | 0.000470664 | 0.780232151 | 0.283 | 0.198 |
| 2 | PA4022 |  | K00138 | 0.002463466 | 0.501204882 | 0.284 | 0.208 |
| 2 | PA0690 |  |  | 0.003426488 | 0.461212646 | 0.355 | 0.279 |
| 2 | PA5349 |  | K05297 | 0.015211542 | 0.65600225 | 0.253 | 0.194 |
| 3 | PA3724 | *lasB* | K01399 | 5.89E-46 | 1.904163151 | 0.345 | 0.11 |
| 3 | PA3049 | *rmf* | K03812 | 7.55E-26 | 1.004662335 | 0.348 | 0.167 |
| 3 | PA5429 | *aspA* | K01744 | 2.59E-21 | 1.281547043 | 0.25 | 0.106 |
| 3 | PA4513 |  | K00380 | 3.19E-12 | 0.626009152 | 0.381 | 0.259 |
| 3 | PA0865 | *hpd* | K00457 | 1.91E-10 | 0.461314675 | 0.457 | 0.332 |
| 3 | PA0872 | *phhA* | K00500 | 1.31E-08 | 0.67425869 | 0.25 | 0.161 |
| 3 | PA0456 |  | K03704 | 5.50E-07 | 0.355236851 | 0.254 | 0.171 |
| 3 | PA4270 | *rpoB* | K03043 | 8.73E-07 | 0.25194872 | 0.442 | 0.345 |
| 3 | PA4554 | *pilY1* | K02674 | 8.99E-07 | 0.451671475 | 0.285 | 0.203 |
| 3 | PA4022 |  | K00138 | 6.77E-06 | 0.420391255 | 0.312 | 0.234 |
| 3 | PA4347 |  |  | 0.000510393 | 0.313914676 | 0.335 | 0.272 |
| 3 | PA0690 |  |  | 0.001014148 | 0.283454513 | 0.312 | 0.251 |
| 3 | PA4236 | *katA* | K03781 | 0.002094632 | 0.202541287 | 0.312 | 0.253 |
| 3 | PA1777 | *oprF* | K03286 | 0.003009983 | 0.179141912 | 0.462 | 0.392 |
| 3 | PA0595 | *ostA* | K04744 | 0.005225118 | 0.183278447 | 0.259 | 0.21 |
| 3 | PA5265 |  |  | 0.019485911 | 0.151210985 | 0.283 | 0.239 |
| 4 | PA3049 | *rmf* | K03812 | 7.41E-17 | 1.53293422 | 0.296 | 0.106 |
| 4 | PA0865 | *hpd* | K00457 | 0.000360956 | 0.4616532 | 0.399 | 0.313 |
| 4 | PA1777 | *oprF* | K03286 | 0.00063926 | 0.559062159 | 0.466 | 0.395 |
| 4 | PA5265 |  |  | 0.001415743 | 0.570791498 | 0.327 | 0.252 |
| 5 | PA4554 | *pilY1* | K02674 | 0.002945229 | 0.804923738 | 0.27 | 0.157 |
| 5 | PA4266 | *fusA1* | K02355 | 0.004106215 | 0.47239558 | 0.31 | 0.192 |
| 5 | PA4513 |  | K00380 | 0.021374521 | 0.528229879 | 0.317 | 0.225 |
| 5 | PA1777 | *oprF* | K03286 | 0.023775314 | 0.653027199 | 0.397 | 0.302 |
| 6 | PA0779 |  | K01338 | 4.50E-08 | 0.739375014 | 0.59 | 0.431 |
| 6 | PA4542 | *clpB* | K03695 | 5.93E-06 | 0.588906871 | 0.455 | 0.305 |
| 6 | PA0865 | *hpd* | K00457 | 9.09E-06 | 0.578710799 | 0.425 | 0.283 |
| 6 | PA4385 | *groEL* | K04077 | 6.10E-05 | 0.432112522 | 0.635 | 0.515 |
| 6 | PA4761 | *dnaK* | K04043 | 7.98E-05 | 0.47758476 | 0.722 | 0.634 |
| 6 | PA5446 |  |  | 0.000405597 | 0.57989457 | 0.316 | 0.211 |
| 6 | PA5349 |  | K05297 | 0.000829255 | 0.513578198 | 0.301 | 0.201 |
| 6 | PA4266 | *fusA1* | K02355 | 0.002226018 | 0.488544938 | 0.323 | 0.231 |
| 6 | PA5054 | *hslU* | K03667 | 0.002249682 | 0.750244042 | 0.308 | 0.223 |
| 6 | PA1596 | *htpG* | K04079 | 0.01664962 | 0.438913772 | 0.35 | 0.27 |
| 6 | PA0709 |  | K11530 | 0.037897515 | 0.442817859 | 0.41 | 0.344 |
| 7 | PA2402 | *pvdI* |  | 5.30E-10 | 1.551166307 | 0.275 | 0.112 |
| 7 | PA3049 | *rmf* | K03812 | 2.98E-05 | 1.002025975 | 0.284 | 0.172 |
| 7 | PA4022 |  | K00138 | 0.000360995 | 0.7391448 | 0.293 | 0.191 |
| 7 | PA5187 |  | K20035 | 0.00100092 | 0.989784605 | 0.263 | 0.178 |
| 7 | PA4513 |  | K00380 | 0.001786944 | 0.637866904 | 0.395 | 0.307 |
| 7 | PA0865 | *hpd* | K00457 | 0.007323569 | 0.495508634 | 0.431 | 0.358 |
| 7 | PA0456 |  | K03704 | 0.037714793 | 0.464332734 | 0.281 | 0.22 |
| 7 | PA5265 |  |  | 0.04609317 | 0.548461558 | 0.302 | 0.256 |
| 8 | PA0865 | *hpd* | K00457 | 3.86E-06 | 0.875758264 | 0.436 | 0.251 |
| 8 | PA3049 | *rmf* | K03812 | 1.39E-05 | 0.9168418 | 0.285 | 0.138 |
| 8 | PA4513 |  | K00380 | 0.005512877 | 0.494966449 | 0.33 | 0.216 |
| 8 | PA0456 |  | K03704 | 0.01280598 | 0.793418636 | 0.257 | 0.173 |
| 8 | PA0997 | *pqsB* | K18001 | 0.027963468 | 0.446217566 | 0.341 | 0.251 |
| 8 | PA4236 | *katA* | K03781 | 0.036327722 | 0.352998692 | 0.358 | 0.267 |
| 9 | PA3724 | *lasB* | K01399 | 9.18458E-15 | 2.041003843 | 0.272 | 0.071 |
| 9 | PA3049 | *rmf* | K03812 | 2.74428E-14 | 2.025508015 | 0.342 | 0.123 |
| 9 | PA4513 |  | K00380 | 1.25274E-05 | 1.022542085 | 0.423 | 0.287 |
| 9 | PA0865 | *hpd* | K00457 | 0.000255237 | 0.666778735 | 0.43 | 0.323 |
| 9 | PA4347 |  |  | 0.000303236 | 0.641968693 | 0.382 | 0.269 |
| 9 | PA5349 |  | K05297 | 0.002745445 | 0.391038889 | 0.268 | 0.174 |
| 9 | PA0690 |  |  | 0.01351193 | 0.598501828 | 0.327 | 0.259 |
| 9 | PA5265 |  |  | 0.02185475 | 0.403363824 | 0.294 | 0.222 |
| 10 | PA2009 | *hmgA* | K00451 | 1.8577E-25 | 1.645245896 | 0.589 | 0.22 |
| 10 | PA3049 | *rmf* | K03812 | 1.22195E-14 | 1.319013028 | 0.394 | 0.154 |
| 10 | PA2402 | *pvdI* |  | 3.74244E-14 | 1.869875418 | 0.271 | 0.076 |
| 10 | PA2008 | *fahA* | K01555 | 3.74871E-13 | 1.142651251 | 0.438 | 0.202 |
| 10 | PA2007 | *maiA* | K01800 | 1.08684E-10 | 1.106818523 | 0.308 | 0.121 |
| 10 | PA4513 |  | K00380 | 5.36487E-09 | 1.075743934 | 0.404 | 0.216 |
| 10 | PA0865 | *hpd* | K00457 | 1.30662E-07 | 0.895043247 | 0.493 | 0.335 |
| 10 | PA4022 |  | K00138 | 0.003349996 | 0.432446907 | 0.284 | 0.195 |
| 10 | PA4236 | *katA* | K03781 | 0.020330934 | 0.47607732 | 0.291 | 0.218 |
| 11 | PA2424 | *pvdL* |  | 5.88504E-12 | 1.141392205 | 0.467 | 0.239 |
| 11 | PA3049 | *rmf* | K03812 | 1.80334E-08 | 1.235492929 | 0.311 | 0.148 |
| 11 | PA2400 | *pvdJ* |  | 2.71077E-07 | 1.227204009 | 0.29 | 0.145 |
| 11 | PA2402 | *pvdI* |  | 5.74301E-05 | 0.635809449 | 0.516 | 0.39 |
| 11 | PA4513 |  | K00380 | 9.54027E-05 | 0.509434201 | 0.425 | 0.299 |
| 11 | PA2393 |  |  | 0.000128159 | 0.783095773 | 0.32 | 0.205 |
| 11 | PA2394 | *pvdN* |  | 0.000368942 | 0.626529719 | 0.263 | 0.16 |
| 11 | PA0865 | *hpd* | K00457 | 0.00219391 | 0.354546009 | 0.418 | 0.302 |
| 11 | PA0456 |  | K03704 | 0.003536143 | 0.4737156 | 0.289 | 0.202 |
| 11 | PA4554 | *pilY1* | K02674 | 0.010403628 | 0.501341085 | 0.305 | 0.227 |
| 11 | PA2386 | *pvdA* | K10531 | 0.014843607 | 0.47237686 | 0.443 | 0.381 |
| 11 | PA2399 | *pvdD* |  | 0.01692865 | 0.63057883 | 0.266 | 0.199 |
| 11 | PA4225 | *pchF* | K12240 | 0.022625328 | 0.532951343 | 0.314 | 0.251 |
| 11 | PA0900 |  |  | 0.032742954 | 0.445942763 | 0.28 | 0.215 |
| 11 | PA1777 | *oprF* | K03286 | 0.034339283 | 0.216173875 | 0.458 | 0.381 |
| 11 | PA0595 | *ostA* | K04744 | 0.035997586 | 0.40406707 | 0.29 | 0.233 |
| 12 | PA5171 | *arcA* | K01478 | 0.00039329 | 0.873485852 | 0.391 | 0.216 |
| 12 | PA5265 |  |  | 0.001106299 | 1.223640385 | 0.276 | 0.132 |
| 12 | PA5172 | *arcB* | K00611 | 0.002232144 | 0.868624671 | 0.397 | 0.251 |
| 12 | PA0139 | *ahpC* | K24119 | 0.02094638 | 0.727607446 | 0.368 | 0.246 |
| **Treatment_36h vs. Control_36h** | **Gene** | **Gene name** | **KEGG annotation** | ***P* value** | **Log2 FoldChange** | **pct.1** | **pct.2** |
| 0 | PA1178 | *oprH* |  | 4.51E-92 | 0.884650181 | 0.848 | 0.687 |
| 0 | PA3552 | *arnB* | K07806 | 5.67E-23 | 0.896396974 | 0.32 | 0.2 |
| 0 | PA1777 | *oprF* | K03286 | 2.10E-13 | 0.408261349 | 0.698 | 0.643 |
| 0 | PA2853 | *oprI* |  | 7.07E-11 | 0.535014681 | 0.363 | 0.283 |
| 0 | PA0865 | *hpd* | K00457 | 5.16E-09 | 0.501338333 | 0.36 | 0.289 |
| 0 | PA2250 | *lpdV* | K00382 | 2.24E-08 | 0.635749023 | 0.261 | 0.197 |
| 0 | PA4442 | *cysN* | K00955 | 5.22E-06 | 0.486385322 | 0.255 | 0.206 |
| 0 | PA4366 | *sodB* | K04564 | 2.00E-05 | 0.452522096 | 0.282 | 0.235 |
| 0 | PA3159 | *wbpA* | K13015 | 4.67E-05 | 0.390301815 | 0.283 | 0.237 |
| 0 | PA4922 | *azu* |  | 0.00016287 | 0.396076369 | 0.291 | 0.25 |
| 0 | PA2247 | *bkdA1* | K00166 | 0.000829779 | 0.422499314 | 0.313 | 0.279 |
| 0 | PA4554 | *pilY1* | K02674 | 0.00119028 | 0.348111666 | 0.473 | 0.451 |
| 0 | PA5078 |  | K03670 | 0.011822412 | 0.409736616 | 0.258 | 0.235 |
| 0 | PA4236 | *katA* | K03781 | 0.021530223 | 0.25712946 | 0.397 | 0.382 |
| 0 | PA5554 | *atpD* | K02112 | 0.024551141 | 0.17916953 | 0.291 | 0.263 |
| 1 | PA1178 | *oprH* |  | 3.47E-41 | 0.981772506 | 0.549 | 0.307 |
| 1 | PA1777 | *oprF* | K03286 | 1.20E-07 | 0.461739293 | 0.306 | 0.217 |
| 2 | PA1178 | *oprH* |  | 8.74E-47 | 0.715580958 | 0.675 | 0.508 |
| 2 | PA3049 | *rmf* | K03812 | 5.47E-23 | 1.058358622 | 0.262 | 0.152 |
| 2 | PA1777 | *oprF* | K03286 | 9.25E-09 | 0.475972712 | 0.41 | 0.346 |
| 2 | PA0865 | *hpd* | K00457 | 1.88E-07 | 0.510261155 | 0.309 | 0.249 |
| 2 | PA2247 | *bkdA1* | K00166 | 0.002606245 | 0.323178391 | 0.289 | 0.257 |
| 2 | PA4554 | *pilY1* | K02674 | 0.017156434 | 0.277677551 | 0.256 | 0.232 |
| 2 | PA4236 | *katA* | K03781 | 0.026632225 | 0.260463662 | 0.284 | 0.259 |
| 3 | PA3724 | *lasB* | K01399 | 3.70E-49 | 2.164203351 | 0.351 | 0.095 |
| 3 | PA1178 | *oprH* |  | 2.12E-37 | 0.976713576 | 0.669 | 0.426 |
| 3 | PA4217 | *phzS* | K20940 | 2.06E-33 | 2.262730075 | 0.254 | 0.07 |
| 3 | PA1777 | *oprF* | K03286 | 1.95E-07 | 0.554276127 | 0.498 | 0.415 |
| 3 | PA4236 | *katA* | K03781 | 5.39E-05 | 0.561308362 | 0.265 | 0.202 |
| 4 | PA1178 | *oprH* |  | 1.89E-40 | 0.902201515 | 0.708 | 0.518 |
| 4 | PA1777 | *oprF* | K03286 | 0.000493527 | 0.42277606 | 0.475 | 0.43 |
| 4 | PA4236 | *katA* | K03781 | 0.00464197 | 0.420879201 | 0.279 | 0.242 |
| 5 | PA1178 | *oprH* |  | 1.67E-26 | 0.636179679 | 0.804 | 0.6 |
| 5 | PA1179 | *phoP* | K07660 | 8.37E-16 | 1.18666765 | 0.299 | 0.154 |
| 5 | PA3552 | *arnB* | K07806 | 1.45E-12 | 0.779337919 | 0.334 | 0.198 |
| 5 | PA1777 | *oprF* | K03286 | 6.13E-06 | 0.533765605 | 0.426 | 0.343 |
| 5 | PA4554 | *pilY1* | K02674 | 0.001936029 | 0.427217335 | 0.26 | 0.203 |
| 6 | PA3690 |  | K01534 | 4.48E-11 | 0.844061748 | 0.511 | 0.366 |
| 6 | PA4623 |  |  | 1.16E-06 | 1.048100369 | 0.263 | 0.161 |
| 6 | PA0139 | *ahpC* | K24119 | 2.77E-06 | 0.931721818 | 0.4 | 0.31 |
| 6 | PA4236 | *katA* | K03781 | 6.55E-06 | 0.896535763 | 0.415 | 0.33 |
| 6 | PA3552 | *arnB* | K07806 | 6.12E-05 | 1.122380367 | 0.264 | 0.184 |
| 7 | PA1178 | *oprH* |  | 4.56E-21 | 0.905763011 | 0.727 | 0.53 |
| 7 | PA1777 | *oprF* | K03286 | 2.22E-05 | 0.603563087 | 0.506 | 0.42 |
| 7 | PA1086 | *flgK* | K02396 | 0.000252125 | 0.683616724 | 0.34 | 0.261 |
| 7 | PA4554 | *pilY1* | K02674 | 0.005528354 | 0.519695762 | 0.344 | 0.29 |
| 7 | PA0865 | *hpd* | K00457 | 0.005993601 | 0.469824863 | 0.303 | 0.246 |
| 8 | PA1178 | *oprH* |  | 5.67E-12 | 0.644974994 | 0.667 | 0.501 |
| 8 | PA0997 | *pqsB* | K18001 | 0.001061412 | 0.600231141 | 0.335 | 0.264 |
| 8 | PA0865 | *hpd* | K00457 | 0.001531656 | 0.542170862 | 0.259 | 0.184 |
| 8 | PA0999 | *pqsD* | K18003 | 0.017131396 | 0.40068628 | 0.342 | 0.293 |
| 9 | PA1178 | *oprH* |  | 5.99E-11 | 0.781235316 | 0.662 | 0.471 |
| 9 | PA1777 | *oprF* | K03286 | 0.000180376 | 0.387572635 | 0.492 | 0.369 |
| 9 | PA3692 | *lptF* |  | 0.000838738 | 0.489310966 | 0.382 | 0.281 |
| 9 | PA4554 | *pilY1* | K02674 | 0.003074604 | 0.383205173 | 0.296 | 0.208 |
| 9 | PA4269 | *rpoC* | K03046 | 0.030662261 | 0.422346698 | 0.285 | 0.227 |
| 10 | PA1178 | *oprH* |  | 2.34E-18 | 0.995092133 | 0.674 | 0.407 |
| 10 | PA1777 | *oprF* | K03286 | 0.001768442 | 0.391133896 | 0.453 | 0.358 |
| 10 | PA4269 | *rpoC* | K03046 | 0.005644405 | 0.486954833 | 0.274 | 0.204 |
| 10 | PA0865 | *hpd* | K00457 | 0.017399608 | 0.461321807 | 0.273 | 0.211 |
| 11 | PA1178 | *oprH* |  | 2.76E-08 | 0.871798321 | 0.696 | 0.484 |
| 11 | PA2402 | *pvdI* |  | 2.91E-06 | 1.267835424 | 0.387 | 0.18 |
| 11 | PA2424 | *pvdL* |  | 6.78E-06 | 1.78798495 | 0.289 | 0.112 |
| 11 | PA1777 | *oprF* | K03286 | 4.15E-05 | 0.888740597 | 0.473 | 0.304 |
| 11 | PA4554 | *pilY1* | K02674 | 0.005087682 | 0.988909852 | 0.328 | 0.211 |
| 12 | PA1178 | *oprH* |  | 0.000428218 | 0.650714855 | 0.693 | 0.516 |

**Table S3** Bacterial strains and plasmids used in this study.

| **Strains and plasmids** | **Relevant characteristics** | **Source** |
| --- | --- | --- |
| Strains |  |  |
| *P. aeruginosa* |  |  |
| PAO1(ATCC15692) | Wild-type | Laboratory stock |
| Δ*pvdA* | *pvdA* deletion mutant in PAO1 | This study |
| Δ*pchD* | *pchD* deletion mutant in PAO1 | This study |
| Δ*pvdA*Δ*pchE* | *pvdA*/*pchE* double deletion mutant in PAO1 | This study |
| PAO1(pME6032) | PAO1 carrying the plasmid pME6032 | This study |
| Δ*pvdA*Δ*pchE*(pME6032-*pvdA*) | Δ*pvdA*Δ*pchE* carrying the plasmid pME6032-*pvdA* | This study |
| Δ*pvdA*Δ*pchE*(pME6032-*pchE*) | Δ*pvdA*Δ*pchE* carrying the plasmid pME6032-*pchE* | This study |
| *E. coli* |  |  |
| DH5α | FΦ80ΔlacZΔM15/Δ(lacZYA-argF)U169recA1endA1hsdR17 | Laboratory stock |
| S17-1 | F-thi pro hsdR [RP4-2 Tc::Mu Km::Tn7 (Tp Sm)] | Laboratory stock |
| Plasmids |  |  |
| pK18*mobsacB* | *sacB*-based gene replacement vector; Km^r^ | Laboratory stock |
| p34s-Gm | Gm resistant cassette carrying vector; Amp^r^ | Laboratory stock |
| pK18-Δ*pvdA*-Gm | Δ*pvdA*::Gm in pK18*mobsacB*; Km^r^, Gm^r^ | This study |
| pK18-Δ*pchD*-Gm | Δ*pchD*::Gm in pK18*mobsacB*; Km^r^, Gm^r^ | This study |
| pK18-Δ*pchE*-Gm | Δ*pchE*::Gm in pK18*mobsacB*; Km^r^, Gm^r^ | This study |
| pME6032 | Shuttle vector containing lacI^q^-Ptac fragment for gene expression;  source of *tetA* gene cassette, Tc^r^ | Laboratory stock |
| pME6032-*pvdA* | *pvdA* was cloned into pME6032 for complementation | This study |
| pME6032-*pchE* | *pchE* was cloned into pME6032 for complementation | This study |

**Table S4** Primers used in this study.

| **Primiers** | **5’-3’ sequence** |  |
| --- | --- | --- |
| *pvdA*-Cy3 | CGGCATGTTCGCGGCTGTAGATGAGATCGG | Sequences of FISH probe primers |
| *pchE*-Cy5 | CATCCGGCACTCCAGCAGCAACAGGGT |  |
| *pvdA*-up-F | CTCGGAATTCCCAGGCTCGGGGATCGAC | To generate pK18-Δ*pvdA*-Gm |
| *pvdA*-up-R | GTGTCGCTGAGCTGCTTGTCCAGGAACAGCAC |  |
| *pvdA*-Low-F | GACAAGCAGCTCAGCGACACCCTGCTGTC |  |
| *pvdA*-Low-R | CTCGTCTAGAGGTCATCGGTCGCGATGG |  |
| *pchD*-up-F | CTCGTCTAGACTCATGATCCGCGGATCG | To generate pK18-Δ*pchD*-Gm |
| *pchD*-up-R | GTATTGCTTCAGGCTCTGGTCCTGCCAGTG |  |
| *pchD*-Low-F | GGACCAGAGCCTGAAGCAATACCTGCACG |  |
| *pchD*-Low-R | CTCGAAGCTTGGCGCTGACTTCCTCGTC |  |
| *pchE*-up-F | CTCGTCTAGAACCCGCGATCTCTACCTC | To generate pK18-Δ*pchE*-Gm |
| *pchE*-up-R | GCTCGCCTCGCGTTCCTGCAGGTACATC |  |
| *pchE*-Low-F | GCAGGAACGCGAGGCGAGCTTCTTCAG |  |
| *pchE*-Low-R | CTCGAAGCTTTCCCGGCCGATACTCAG |  |
| *pvdA* F | GTCCGAGCTCATGACTCAGGCAACTGCAAC | To generate pME6032-*pvdA* |
| *pvdA* R | ACCGCTCGAGGTGGCGCCGATCAGCTGG |  |
| *pchE* F | GTCCGAGCTCATGGATCTGCCCCCCGATTC | To generate pME6032-*pchE* |
| *pchE* R | ACCGCTCGAGCTCATAGCACGCCCTCTTCC |  |
